# An Integration of Deep Neural Network-Based Extended Kalman Filter (DNN-EKF) Method in Ultra-Wideband (UWB) Localization for Distance Loss Optimization

**DOI:** 10.3390/s24237643

**Published:** 2024-11-29

**Authors:** Chanthol Eang, Seungjae Lee

**Affiliations:** Department of Computer Science and Engineering, Intelligent Robot Research Institute, Sun Moon University, Asan 31460, Republic of Korea; ngchanthol1@gmail.com

**Keywords:** deep neural networks (DNNs), extended Kalman filter (EKF), ultra-wideband (UWB) technology, deep neural network-based extended Kalman filter (DNN-EKF)

## Abstract

This paper examines the critical role of indoor positioning for robots, with a particular focus on small and confined spaces such as homes, warehouses, and similar environments. We develop an algorithm by integrating deep neural networks (DNNs) with the extended Kalman filter (EKF) method, which is known as DNN-EKF, to obtain an accurate indoor localization for ensuring precise and reliable robot movements within the use of Ultra-Wideband (UWB) technology. The study introduces a novel methodology that combines advanced technology, including DNN, filtering techniques, specifically the EKF and UWB technology, with the objective of enhancing the accuracy of indoor localization systems. The objective of integrating these technologies is to develop a more robust and dependable solution for robot navigation in challenging indoor environments. The proposed approach combines a DNN with the EKF to significantly improve indoor localization accuracy for mobile robots. The results clearly show that the proposed model outperforms existing methods, including NN-EKF, LPF-EKF, and other traditional approaches. In particular, the DNN-EKF method achieves optimal performance with the least distance loss compared to NN-EKF and LPF-EKF. These results highlight the superior effectiveness of the DNN-EKF method in providing precise localization in indoor environments, especially when utilizing UWB technology. This makes the model highly suitable for real-time robotic applications, particularly in dynamic and noisy environments.

## 1. Introduction

Indoor positioning technologies are essential for determining the location of objects within enclosed spaces, including offices, hospitals, restaurants, and warehouses. These technologies are widely used to track a variety of entities, including people and robots, within buildings, with the primary objective of obtaining accurate spatial information. This information is essential for optimizing processes, improving operational efficiency, and ensuring safety. In environments such as factories or warehouses, where operational efficiency is of the utmost importance, indoor positioning systems have become a standard feature. They provide valuable data that can be used to streamline workflows, reduce downtime, and enhance overall productivity. Nevertheless, achieving precise indoor positioning remains a significant challenge due to the intricate nature of indoor environments. These spaces are often dynamic, with frequent movement of people, machinery, and other objects, which can present a challenge for accurate indoor positioning. Furthermore, indoor environments are often congested with a variety of obstacles, including walls, furniture, and equipment made from different materials, which can impede signal transmission. These factors contribute to difficulties in obtaining precise positioning data, as they introduce a range of problems, including signal blockage, line-of-sight obstructions, shadowing, multi-path effects, and electromagnetic interference [1,2]. The specific characteristics of each indoor environment can vary significantly, making it challenging to implement a universal solution that will work in all cases. Many existing studies have explored various technologies to overcome these challenges in indoor localization. Some of these approaches include the use of ultrasonic sensors, Ultra-Wideband (UWB) technology, and other sensor-based systems to improve the accuracy of indoor positioning. While these technologies have shown promise, they often fall short of achieving the desired level of precision in all environments due to the inherent complexities mentioned [3,4,5,6].

UWB technology represents a state-of-the-art wireless communication method that transmits data using extremely short pulses, often measured in microseconds or even nanoseconds. One of the most significant advantages of UWB technology is its extensive frequency range, which enables it to transmit data over long distances with high speed and low power consumption. UWB is particularly notable for its resilience to interference, making it an ideal solution for environments where multiple signals might overlap or where traditional communication methods are prone to noise and signal degradation. These characteristics make UWB technology an ideal solution for a wide range of applications, including precise positioning and ranging, data exchange, and object detection using radar [7,8]. In the field of positioning and ranging, UWB’s capacity to deliver high-resolution distance measurements is particularly advantageous, as it can attain centimeter-level precision in locating objects or individuals within an indoor environment. This level of precision is essential for applications such as indoor navigation, asset tracking, and enhanced security systems, where accurate real-time location data is crucial. However, while UWB technology offers impressive capabilities, it is not a standalone solution for achieving the highest levels of localization accuracy required in complex and dynamic indoor environments. Integrating UWB-based positioning systems with artificial intelligence and advanced filtering methods can further enhance their accuracy. Artificial intelligence techniques, such as deep neural networks (DNNs), can be employed to analyze and interpret the raw data generated by UWB sensors, identifying patterns and correcting errors that may arise due to signal interference or environmental factors. Similarly, filtering methods like the extended Kalman filter (EKF) help to refine the position estimates by smoothing out the noise and compensating for any inaccuracies in the measurement process. The integration of UWB technology with AI and filtering methods represents a promising avenue for enhancing the precision and reliability of location data in complex indoor environments.

The integration of artificial intelligence and advanced filtering methods is essential to further enhance the accuracy of UWB technology. While UWB technology offers a robust foundation for precise positioning, the complexities of indoor environments, including signal multipath effects, reflections, and interference, require additional processing to achieve the highest levels of precision. To overcome these challenges, a combination of techniques is often used. DNNs are highly effective at learning complex patterns from the raw data generated by UWB sensors, enabling the system to make more accurate predictions about object locations. Neural networks can be trained to recognize and compensate for environmental factors that might otherwise introduce errors into the localization process, thus ensuring more accurate results. In addition to DNNs, a variety of filtering techniques are essential for refining positioning estimates. The Kalman filter is a widely used tool in signal processing, enabling the prediction of a system’s state over time. It effectively reduces noise and uncertainty in measurements. The extended Kalman filter (EKF) extends this capability to nonlinear systems, making it suitable for more complex indoor environments where the relationship between measurements and positions is not straightforward [9,10,11,12]. Another crucial technique is the moving average filter, which helps to mitigate the impact of sudden fluctuations or outliers in the data by averaging a series of measurements over time. This results in a more stable and reliable position estimate, especially in scenarios where the signal quality might be inconsistent due to obstacles or interference [13,14]. To address the challenges of indoor localization, this paper presents a novel approach integrating DNNs with the EKF method, known as the deep neural network-based extended Kalman filter (DNN-EKF) method. The DNN-EKF algorithm offers a significant advancement in indoor localization, which outperforms the current existing works from references [15,16] discussed in this paper as well as the current existing work of integrating the low-pass filter and extended Kalman filter (LPF-EKF). Using UWB technology to facilitate precise and reliable robot movements can ensure accurate localization in complex indoor environments.

There are many methods for enhancing indoor localization to ensure precise and reliable robot movements by integrating with various deep learning models. However, in this work, we integrate the EKF model with the DNN model, which represents our primary proposed model in this paper. The combination of the two models results in an enhancement in indoor positioning for robots. The EKF is highly effective at fusing data from multiple sensors, reducing noise through probabilistic filtering, and predicting the robot’s state. It continuously corrects estimates in real time as new sensor data becomes available, making it suitable for both linear and nonlinear environments. Integrating the EKF with a DNN enhances localization accuracy by leveraging the DNN’s ability to model complex relationships and noise patterns in sensor data, which refines the EKF’s estimates. This combination results in an adaptive and robust system that can dynamically adjust to varying indoor environments, compensate for sensor drift, and remain resilient to sensor failures. Consequently, it ensures precise and reliable robot movements, making it ideal for challenging indoor scenarios. Integrating the EKF and DNN for indoor localization combines the strengths of both models, providing a more robust and accurate approach for indoor positioning for robots, with the following benefits:

- Improved sensor fusion accuracy: The DNN’s capability to model nonlinearities enhances the EKF’s sensor fusion accuracy, leading to more precise localization.
- Enhanced noise filtering: While the EKF reduces general sensor noise, a DNN can learn specific noise patterns, making the EKF’s filtering more effective.
- Dynamic adaptation to environments: A DNN can adaptively update the system models based on real-time and historical data, allowing the EKF to adjust to changing indoor conditions dynamically.
- Increased robustness to sensor failures: A DNN can estimate missing sensor values, allowing the EKF to maintain accurate localization even with partial sensor data loss.
- Lower computational load with higher accuracy: A DNN can preprocess and filter sensor data, allowing the EKF to focus on refined data, reducing computational costs while improving accuracy.
- Reliable predictions for motion planning: DNN-based predictions of future positions can be used by the EKF for accurate motion planning, ensuring smoother and safer robot movements.
- Adaptive correction model: With a DNN’s learning capability, the EKF’s correction model can be fine-tuned based on past errors, leading to increasingly precise localization as more data becomes available.

The remainder of this paper is structured as follows: Section 2 presents the related work concerning various models of indoor localization. Section 3 presents the proposed methodology, which will cover the current existing work of the LPF-AF, LPF-KF, LPF-EKF, NN-EKF, and the proposed DNN-EKF methods. The proposed solution flowchart and algorithm are also included in Section 3. Section 4 presents the simulation settings, parameters, and the existing work on indoor localization. It also explains the experimentation of the proposed algorithm and its result. Finally, a conclusion of our work contributions and the significant task for future research are presented in Section 5.

## 2. Related Work

In the field of indoor localization, the integration of UWB technology with advanced filtering techniques, including Kalman filters, EKFs, and neural networks, has emerged as a key area of focus for enhancing accuracy and reliability. The research in this area has demonstrated that the combination of these methods can markedly enhance the precision of location estimation in complex indoor environments. For instance, Borhan et al. (2023) [15] put forth a hybrid methodology that integrates the Kalman filter with the moving average (MA) filter to enhance the precision of location estimation through the use of UWB technology. The efficacy of the proposed method was evaluated through a comparative analysis with the conventional Kalman filtering approach. The findings revealed that the proposed strategy exhibited superior performance in reducing errors, exceeding the capabilities of the traditional Kalman filter. The hybrid method’s capacity to minimize localization errors renders it a valuable benchmark for comparison in the proposed work. Similarly, Karfakis et al. (2022) [16] investigated two distinct methodologies for forecasting UWB position values. The initial method entails the utilization of EKF techniques to ascertain the robot’s trajectory, capitalizing on the EKF’s performance to address nonlinearities inherent to the localization process. The second approach employs a neural network to model the relationship between positional errors in UWB data and signal quality metrics, such as the estimate of precision and received signal strength. The findings indicate that the neural networks are particularly effective at representing sensor covariance and dynamically adjusting the reliability of EKF estimates. This adaptive capability enables the neural networks to compensate for data loss by utilizing alternative estimation sources, when necessary, thereby enhancing the robustness of the localization system. The aforementioned methods, namely Borhan et al.’s filtering approach and Karfakis et al.’s neural network-based EKF (NN-EKF) integration, serve as crucial comparative benchmarks for our proposed DNN-EKF algorithm. By comparing our approach against these established methods, we aim to demonstrate the superior accuracy and reliability of our solution in achieving precise indoor localization.

In a recent study, Cano et al. (2023) [17] proposed an adjustment to an empirical variance model that was specifically designed for UWB range measurements, which are derived from time-of-flight (ToF) estimates. This adjustment employs the received first path power (FPP) to enhance accuracy. Building upon this work, they developed a robust and resilient M-estimation robust Kalman filter (M-RKF) to create a sophisticated navigation system based on UWB technology capable of effectively managing multipath (MP) outliers that often compromise measurement reliability. Through comprehensive experimental verification using real-world data, they successfully demonstrated the advantages and efficacy of both our enhanced variance model and the M-RKF in environments where multipath interference is pervasive. In [18], the authors put forth an algorithm that employs UWB technology to achieve relative localization without the need for a direct line of sight to the target, thereby broadening the scope of potential applications for UWB in more intricate settings. Furthermore, in [19], a novel localization framework was proposed, which employs the transmission signals from a mobile UWB sensor positioned outside a building. Subsequently, the received signals are subjected to meticulous analysis in accordance with the modified Saleh–Valenzuela (SV) channel model, thereby offering a novel approach to UWB-based localization in challenging scenarios. In a study by Kulikov et al. (2020) [20], they proposed an algorithm for an EKF that employs the use of cost-effective UWB radio systems for range measurements. This approach is designed to enhance the precision of indoor localization by employing a system comprising fixed base stations, or anchors, with known positions and tags that serve as the tracked points. The algorithm effectively integrates the range measurements obtained from these UWB radio systems, thereby enabling precise location estimation even with affordable hardware components. This renders the approach especially appealing for applications where budgetary limitations are a significant consideration. Further exploration in this area was conducted in studies [21,22], in which researchers compared various centralized Kalman filter algorithms for indoor positioning within a wireless sensor network. The objective of their research was to evaluate the performance of various Kalman filter approaches, including EKF and the Unscented Kalman Filter (UKF), in a hybrid indoor localization system. The objective of the studies was to ascertain which filtering method offers the optimal balance between computational efficiency and localization accuracy in scenarios involving complex indoor environments with multiple sensors.

The objective of journal article [23] is to develop a precise indoor positioning system that employs the integration of Support Vector Machine (SVM) and Long Short-Term Memory (LSTM) algorithms to enhance location accuracy within indoor environments. The study employs the SVM for its robust classification capabilities and LSTM for its strength in handling sequential data and temporal dependencies. The objective of integrating these algorithms is to surmount the prevalent challenges associated with indoor localization, such as signal variability and interference, and thereby facilitate dependable and precise location tracking. The approach was evaluated through experiments and demonstrated to offer improved performance compared to traditional methods, thereby representing a significant advancement in indoor positioning technology. In [24], the author proposed an innovative approach to indoor positioning by applying an SVM to data gathered from wireless sensor networks. The research underscores the potential of the SVM, renowned for its robust classification capabilities, to accurately determine a device’s location within a building by analyzing sensor data such as signal strength, signal-to-noise ratios, or time-of-flight measurements. The study outlines the development and implementation of an SVM-based model that processes these inputs to classify and predict the device’s position with high precision. Through experimental evaluations, the paper demonstrates that the SVM-based method significantly enhances localization accuracy compared to traditional approaches, which often encounter difficulties with issues such as signal interference and environmental variability. This advancement has practical implications for improving navigation systems, asset tracking, and various indoor applications where accurate location data are crucial.

Next, according to [25], the authors introduced a method to enhance the accuracy of indoor localization for mobile robots by employing an EKF in conjunction with multiple data sources. The research presents a method for integrating various sensor inputs, including odometry, GPS, and inertial measurements, through the use of an EKF, which is a statistical algorithm employed for the estimation of the state of a dynamic system. The objective of the approach is to enhance the robustness and precision of the robot’s localization within indoor environments by integrating disparate data streams. This integration addresses challenges such as sensor noise and drift. The study demonstrates the efficacy of the EKF-based fusion technique in refining the robot’s position and orientation estimates, thereby enhancing the reliability of navigation and obstacle avoidance. The paper presents experimental evidence demonstrating the efficacy of this method in enhancing the performance of indoor localization for mobile robots, thereby making a valuable contribution to the field of autonomous navigation technology. In [26], the authors introduced a positioning algorithm that fuses data from an Inertial Measurement Unit (IMU), Ultra-Wideband (UWB) sensors, and an odometer to enhance the accuracy of indoor localization. The algorithm is based on the EKF, and it is used to combine data from the aforementioned diverse data sources. Each of these sources has its own strengths and limitations: the IMU is used for motion tracking, UWB for precise distance measurements, and the odometer for velocity estimation. By integrating these inputs through the EKF, the algorithm enhances the reliability and accuracy of position estimates, thereby mitigating issues such as sensor drift, noise, and signal interference. The study demonstrates that this fusion approach markedly enhances the precision of indoor positioning systems, particularly in environments where individual sensors may be prone to limitations. The results demonstrate the efficacy of the EKF in developing a robust and accurate localization solution for applications such as autonomous vehicles and robotics in indoor settings. Based on [27], the authors provided a detailed account of the development of a comprehensive indoor positioning system for a greenhouse robot, which employs a fusion of UWB sensors, an Inertial Measurement Unit (IMU), an odometer (ODOM), and LiDAR. The objective of integrating these technologies is to provide precise and reliable localization in the challenging environment of a greenhouse, where factors such as dense vegetation and varying light conditions can disrupt traditional positioning methods. UWB technology enables precise distance measurement, while an IMU facilitates motion and orientation tracking. The odometer provides velocity estimation, and light detection and ranging (LiDAR) enables the mapping of the surrounding environment to detect obstacles. The integration of these data sources enables the system to enhance the robot’s capacity for autonomous navigation and operation within the greenhouse environment. The study demonstrates that this multi-sensor approach significantly improves localization accuracy, thereby ensuring efficient and effective automation for tasks such as planting, monitoring, and harvesting in agricultural settings.

In [28], the researcher presented a novel localization system designed for service robots operating in challenging indoor environments, such as those with low lighting and slippery surfaces. The system integrates data from focal optical flow sensors with other sensor inputs through the application of sensor fusion techniques, thereby enhancing the robot’s capacity to accurately determine its position and navigate in conditions where traditional methods may prove ineffective. The optical flow sensor is utilized to track motion by detecting alterations in visual patterns, whereas sensor fusion serves to enhance reliability by integrating these data with other measurements. This approach addresses issues such as poor lighting and surface slipperiness, thereby ensuring that the robot can maintain accurate localization and stable movement. In [29], the researcher assessed and characterized the uncertainties inherent to various mobile robot localization techniques. By employing high-precision optical surveying instruments, the study systematically evaluates the accuracy and reliability of disparate localization methods, identifying sources of error and quantifying their impact on the robot’s positioning accuracy. This characterization elucidates the limitations of current localization technologies and provides insights into enhancing their precision, particularly in environments where accurate localization is critical for the robot’s performance. According to [30], the authors introduced a case study on the improvement of algorithms for robot positioning and navigation using Simultaneous Localization and Mapping (SLAM). Simultaneous Localization and Mapping (SLAM) enables a robot to construct a map of an unmapped environment while simultaneously tracking its position within that environment. The study enhances SLAM algorithms to improve accuracy, address challenges such as sensor noise and environmental changes, and enhance computational efficiency. Finally, in a recent study, Ranjan et al. (2024) [31], the researchers examined several proposed filtering techniques with the aim of enhancing the accuracy of UWB localization in indoor settings. These include the low-pass filter and Kalman filter (LPF-KF), the low-pass filter and extended Kalman filter (LPF-EKF), and the low-pass filter and moving average filter (LPF-AF). The findings of this study will be used as a basis for comparison in our proposed DNN-EKF model. The numerical results demonstrate that our model outperforms the models in terms of optimizing distance loss as well as improving localization accuracy.

## 3. Proposed Methodology

This section describes an overview of the architectural design of the UWB system, the vision tracking system, and the Robot Operating System (ROS) ecosystem. The UWB system’s architectural design is intended to facilitate the positioning and localization of robots. To ascertain the veracity of the data, the vision tracking system employs camera vision techniques to track and monitor the robot’s movement. ROS 1.15.14 Noetic is a software framework that facilitates communication between software and hardware, providing tools for the construction, testing, and deployment of localization solutions.

### 3.1. System Architecture

**Figure 1:**
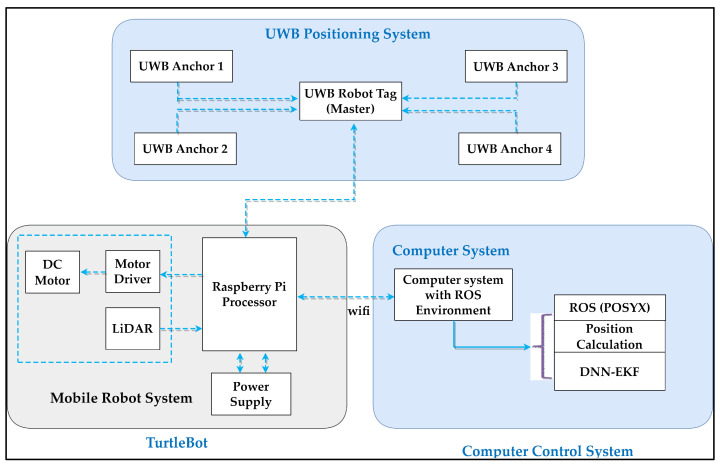
System architecture of the proposed model.

As illustrated in Figure 1, the robot positioning system comprises a UWB positioning subsystem, a remote computer, and a mobile robot. The UWB positioning subsystem comprises UWB anchors that are fixed within the environment and a UWB tag attached to the robot [32]. The computer executes positioning algorithms to ascertain the robot’s position and coordinates by measuring the distance between the UWB tag and the anchors. The remote computer is in communication with the robot via a wireless connection. It processes the data obtained from the positioning system, calculates the coordinates of the robot, and controls its movements. The system is designed to accommodate a range of tasks, including interactive communication, robot control, position estimation, and the display of the robot’s position. However, due to the potential for error or instability, relying on a single sensor may not achieve the desired level of accuracy. The integration of UWB tags with anchor sensors enhances positioning accuracy and stability. To further improve accuracy, this study proposes a position estimation and error correction method based on the EKF algorithm.

As illustrated in Figure 1, the robot positioning system is capable of simultaneously acquiring and integrating data from both UWB tags and anchors, ensuring more precise and reliable position estimation. The proposed system architecture comprises three principal components: the UWB positioning system, the mobile robot, and the computer control systems. The initial component comprises several UWB tags and anchors. The second component is the robot development system, specifically a mobile robot system, with TurtleBot 3 utilized for the preliminary position experimentation. The system comprises a motor for locomotion, a driver unit for motor control, and a LiDAR sensor for scanning and obstacle detection, all of which are powered by a Raspberry Pi processor. In conclusion, the computer control system, equipped with an ROS environment and the POZYX library, communicates with the Raspberry Pi, which serves as a read/write device. A variety of algorithms can be tested and simulated with the objective of enhancing localization and positioning. TurtleBot 3 is equipped with UWB tag nodes that are compatible with the ROS ecosystem. The connection between the UWB robot tag and the Raspberry Pi is established via a USB interface, as indicated by the dashed line. The sensor measurements, in conjunction with the wheel odometry and the output from the LPF algorithm, are employed in the EKF for localization purposes. The LiDAR-based navigation stack is initiated with UWB ranging and LiDAR scanning. Moreover, the TurtleBot 3 ROS library enables the creation of a simulation environment.

### 3.2. Ultra-Wideband (UWB) Technology System

UWB technology, traditionally utilized in wireless communications, has recently gained significant traction in positioning applications due to its unique properties. The wide bandwidth of UWB not only prevents interference from other radio frequency signals but also enables effective signal penetration through obstacles such as walls and other barriers. This characteristic renders UWB particularly reliable in challenging environments, including non-line-of-sight (NLOS) and multipath scenarios where direct signal paths are often obstructed [32]. Furthermore, the automatic identification of tags within a UWB system effectively streamlines data association challenges, thereby reducing potential errors in multi-tag environments. With regard to distance determination, UWB technology functions by transmitting ultra-short radio signals from a mobile transceiver (tag) to a set of known anchors, measuring the time of flight (ToF) of these signals, and subsequently calculating precise distances based on these measurements. This high level of precision in distance measurement serves to enhance the accuracy of UWB-based positioning systems, rendering them suitable for a diverse array of applications, including indoor navigation, asset tracking, and autonomous robotics.

The study employs the POZYX system, a sophisticated UWB-based hardware solution designed for precise position and motion sensing. The system permits the configuration of UWB settings according to four key parameters that directly influence system performance. Notwithstanding the sophisticated settings described above, the presence of noise and uncertainty in the data collected by UWB sensors can still present challenges to achieving accurate position estimation. To address these issues, the study incorporates advanced optimization techniques, including the EKF and deep learning models. These methods are of great benefit in reducing the impact of measurement noise and improving the accuracy of indoor localization systems that rely on UWB technology. The objective of the study is to enhance the precision and reliability of position and motion sensing in complex indoor environments by leveraging advanced algorithms. As illustrated in Figure 1, the robot positioning system comprises a UWB positioning subsystem, a remote computer, and a mobile robot. The UWB positioning subsystem comprises fixed UWB anchors situated within the environment, as well as a UWB robot tag [33]. By employing distance measurements between the UWB tag and the anchors, the computer executes positioning algorithms to ascertain the exact position and coordinates of the robot. A wireless communication link connects the remote computer to the robot, allowing for data processing, coordinate calculation, and motion control.

The system serves multiple functions, including interactive communication, robot control, position estimation, and robot position display [34]. Recognizing that a single sensor may not achieve high accuracy due to errors or instability, the combined usage of UWB tags and anchor sensors is implemented to enhance positioning accuracy and stability. This study proposes a robust method for position estimation and error correction, which integrates a DNN with an EKF algorithm. This approach capitalizes on the respective strengths of both techniques, thereby enhancing accuracy and reliability. The robot positioning system can acquire and integrate data from UWB tags and anchors in real time, effectively managing the noise and uncertainties inherent in the measurements. By integrating the predictive capabilities of the EKF with the adaptive learning of the DNN, the system can achieve a high level of precision and robustness in a variety of operational environments, demonstrating enhanced performance in complex and dynamic conditions.

The distribution of UWB nodes plays a crucial role in determining the accuracy of indoor positioning for mobile robots, such as the TurtleBot 3, by providing high-precision range measurements based on time of flight (ToF). The accuracy of localization depends on factors like the density and placement of UWB nodes. A higher density of nodes leads to better coverage and more frequent, reliable distance measurements, improving the robot’s position estimate, especially in environments with limited line-of-sight characteristics. Proper placement of nodes minimizes gaps and blind spots, reducing uncertainties in the robot’s position. In this work, incorporating UWB nodes within the DNN-EKF framework significantly enhances localization accuracy. UWB provides precise range measurements that serve as valuable input for the DNN component, allowing the system to learn patterns from the data. When combined with the EKF, UWB data, along with other sensor data, help produce accurate real-time state estimates, improving the robot’s position and velocity predictions. This integration of UWB nodes into DNN-EKF results in more reliable and resilient indoor localization, especially in challenging environments where traditional sensors may struggle.

### 3.3. Experimental Environment for UWB System Localization

**Figure 2:**
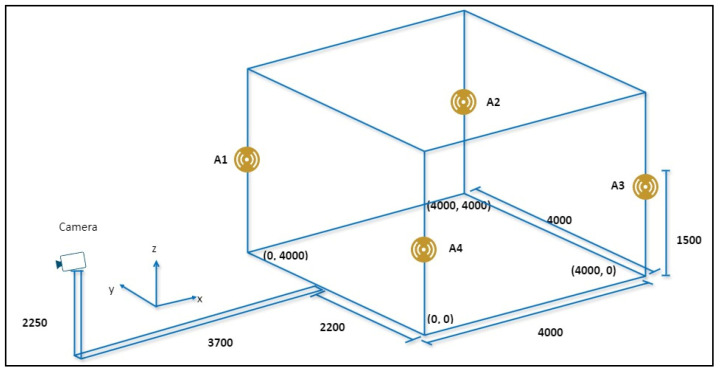
Experimental environment with visual tracking system.

A visual tracking system is used to track and estimate the motion of objects in a sequence of images or video frames captured by a webcam within the experimental environment involving the UWB system. Visual tracking is a fundamental task with a wide range of applications across various fields, including surveillance, robotics, augmented reality, and human–computer interaction [31]. As illustrated in Figure 2, visual tracking methodologies are devised to ensure the accurate and robust localization and tracking of objects of interest over time, even in the presence of considerable challenges. These challenges include changes in the object’s appearance, variations in scale, alterations in orientation, and partial or complete occlusion by other objects. Effective visual tracking systems must overcome these obstacles to provide reliable data, ensuring that the tracked objects are continuously monitored and analyzed. This is crucial for maintaining the accuracy and efficiency of applications that rely on real-time object tracking and motion estimation.

Figure 2 illustrates a fundamental process for visual tracking, which was conducted using a webcam that was installed and tested at the IRRI laboratory of Sun Moon University in South Korea. The webcam was positioned in an optimal location, ensuring that all quadrants of the tracking area on the webcam were within the camera’s field of view. Based on the data obtained from the webcam, four positions were captured and saved in a two-dimensional projected coordinate system. A perspective transformation matrix was constructed using the saved positions, with the objective of mapping the webcam’s view to the desired tracking area. Subsequently, the image matrix and perspective transformation matrix were calculated using a resolution of 512 × 512, with the objective of achieving optimal tracking performance. The visual tracking process began after applying perspective transformation [35]. We selected a high-contrast area of the robot for tracking. An appropriate tracking algorithm, such as the Discriminative Correlation Filter with Channel and Spatial Reliability (CSRT) algorithm, is implemented to track the robot within the 512 × 512 images [36]. The tracking data provide the position within the 512 × 512 image and are subsequently converted to the corresponding position in the real environment, which may have different dimensions. By scaling the tracked position using Equation (1), the position is mapped to a 4000 mm × 4000 mm environment in the x- and y-coordinates, where $\bar{x}$ and $\bar{y}$ represent the positions within the 512 × 512 image.

(1) $$x=\frac{\bar{x}}{512}\times 4000,\;y=\frac{\bar{y}}{512}\times 4000\;$$

This systematic and comprehensive approach highlights the significance of webcam calibration, perspective transformation, and precise scaling in the development of a robust visual tracking system. The incorporation of these sophisticated techniques enables the system to not only monitor the robot’s movement but also to do so with enhanced accuracy, rendering it well suited for applications where precision is of paramount importance. Furthermore, although the UWB node-based source localization algorithm is highly effective at determining the position of a robot, it is deficient in providing the robot’s orientation in relation to a map. To address this limitation, we propose an approach that integrates a map with a LiDAR scan matching technique. This integrated approach is essential for resolving orientation ambiguity by aligning the robot’s observed LiDAR scans with a pre-existing map, thereby determining the robot’s precise heading. Once the initial heading is accurately estimated, the robot’s pose, including its orientation, is published to the initial pose topic. This integration enhances not only the localization accuracy but also provides a comprehensive understanding of the robot’s spatial orientation within the mapped environment, facilitating more precise navigation and interaction with its surroundings.

**Figure 3:**
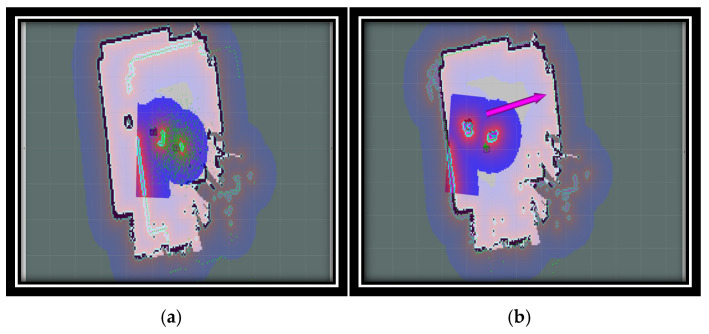
Robot poses and LiDAR scans before the automatic initialization (**a**); robot poses and LiDAR scans after the automatic initialization (**b**) [31].

Figure 3 provides a visual representation of the contrast in the robot’s pose before and after the initialization process. Prior to autonomous initialization, the robot’s pose is represented with a high degree of inaccuracy, resulting in a noticeable disparity between the scan data and the actual map, as illustrated in Figure 3a. In contrast, following initialization, the scan data are aligned seamlessly with the map, as demonstrated in Figure 3b.

Once the initial position of the robot has been established, it commences movement toward each specific point, utilizing the move-base algorithm within the ROS navigation stack. Subsequently, the final location of the robot is determined on the map and in the physical environment, following a meticulous initialization process. The precision of the robot’s arrival at its intended destination is corroborated through a comprehensive examination of LiDAR scans superimposed on the map, thereby confirming that the robot’s movements are exact and dependable.

### 3.4. Simulation Tool Within the ROS Ecosystem

This section will examine the ROS ecosystem and its components, including nodes, topics, and messages, as well as the integration of UWB sensors into this framework. Furthermore, the discussion will address the manner in which UWB sensors quantify the distance between a robot and its surrounding environment and the extent to which these measurements can augment the robot’s localization and mapping capabilities. In the context of navigation systems, the accuracy of position data is of paramount importance. UWB sensors are particularly well suited for robot localization because they provide low-noise range measurements that are resistant to multipath interference. This renders them an optimal selection for challenging environmental conditions. The integration of UWB data with odometry information provides a robust solution for accurate localization. The POZYX system employs UWB technology to attain centimeter-level precision, thereby exceeding the accuracy of conventional Wi-Fi and Bluetooth positioning systems. The system’s algorithm employs the Kalman filter to compute the robot’s position. This incorporates odometry data to establish the motion model and updates the robot’s pose using UWB range measurements. The graphical user interface (GUI) displays both the map and the current robot position. Additionally, the navigation stack can be supplied with Kalman filter-based pose information as needed. A number of ROS packages are available for the collection and processing of IMU sensor data in the context of mobile robotics. To illustrate, the ROS IMU package offers an implementation of an IMU sensor driver and a filter for estimating the robot’s orientation based on sensor data. Furthermore, the robot localization package provides an implementation of the EKF for the fusion of data from multiple sensors, including IMU data, with the objective of estimating the robot’s position and orientation.

We utilized RViz, a three-dimensional visualization tool, to display a range of elements, including sensor data, robot models, and robot states. Figure 3a illustrates a map generated from sensor data collected by the robot. In this map, the white dots form shapes that are likely representative of obstacles or objects detected by the robot’s sensors. Green lines indicate specific paths or directions related to the robot’s movement or sensor orientation. Subsequent to the experiment illustrated in Figure 3b, a more comprehensive map was generated, featuring colored regions in purple, white, and green. Two green circles with red lines, which may signify orientation, represent the robot or its sensors. A purple arrow extending from one of the green circles is indicative of a planned path or direction of movement. The background grid indicates that this visualization is intended to serve as a spatial mapping or planning tool. The RViz tool facilitated effective data visualization and analysis, enabling the presentation of complex data in a manner that facilitated comprehension and interpretation.

### 3.5. Existing Research and Proposed Localization Algorithms

Our work will be compared to the recent research presented in [16,31], which focuses on UWB-aided mobile robot localization with neural networks and the EKF, and the comparative analysis of integrated filtering methods using UWB localization in indoor environments. To demonstrate the effectiveness of our proposed DNN-EKF model, the algorithms from these two studies will be examined and experimentally tested in our work as part of a comparative analysis.

#### 3.5.1. Extended Kalman Filter (EKF)

This section presents an overview of the extended Kalman filter (EKF) algorithm and provides an illustrative solution for implementing the filtering process. The EKF is designed to extract pertinent information from noisy or incomplete data by applying sophisticated mathematical techniques. The EKF is a widely utilized algorithm for localization in robotics, navigation, and autonomous systems. It extends the traditional Kalman filter to address nonlinear system states [37,38,39]. The EKF operates recursively, estimating the system’s state over time by incorporating nonlinear functions into a linear approximation, which is then updated using a recursive Bayesian filter. The algorithm employs a set of linearized system models and measurements to estimate the system state, taking into account both model uncertainty and measurement noise. The operation involves two main steps: prediction and correction. During prediction, the EKF forecasts the system state at the next time step based on the present state and control inputs. In the correction step, the algorithm refines the prediction using the most recent measurement. Renowned for its proficiency in handling nonlinear systems and providing accurate estimates amid measurement noise, the EKF finds extensive application in diverse fields such as robotic navigation and autonomous systems. Its computational efficiency further renders it suitable for real-time applications.

- Prediction step:

In the prediction step (estimation equations), the prior estimated state can be expressed as follows [21]:

(2) $$x_{k}=A_{k}x_{k-1}+w_{k,}w_{k}\sim N(0,Q)$$

(3) $$z_{k}=f\left(x_{k},v_{k}\right),v_{k}+r_{k}\sim N(E(e_{r}),R)$$

First, the observation value, $z_{k}$, is the vector of ranges ${\left(r_{1},r_{2},r_{3},\ldots \right)}^{T}$. Noise vector $v_{k}$ follows the Gaussian distribution according to Equation (2). Four anchors are located at $\left(x_{1},\;y_{1}\right)$, $\left(x_{2},\;y_{2}\right)$, $\left(x_{3},\;y_{3}\right)$, and $\left(x_{4},\;y_{4}\right)$, and $z_{k}$ can be expressed as

zk=r1r2r3r4=fxk+vk.

(4) =f1(Xk,Yk)f2(Xk,Yk)f3(Xk,Yk)f4(Xk,Yk)+vk.

(5) vk=E1E2E3E4+δ1δ2δ3δ4.

where k is the Kalman gain that minimize the measurement covariance, and the measurement error that follows the Gaussian distribution ($e_{r}$) can be expressed as

(6) $$e_{r}=E\left(e_{r}\right)+\delta ,\delta \sim N\left(0,\sigma_{r}^{2}\right).$$

In the prediction step of the EKF, the filter forecasts the subsequent state of a dynamic system by applying the system’s dynamic model and incorporating the nonlinear transition function to account for nonlinearity. This process entails the prediction of both the state and its associated covariance matrix, which captures the uncertainty inherent in the state prediction. This is achieved by considering the impact of system dynamics and external factors. The incorporation of process noise, represented by a zero-mean Gaussian random variable, addresses the inherent uncertainty associated with the dynamic model. In linear systems, a transition matrix is employed to propagate the state and covariance matrix, while in nonlinear systems, the Jacobian matrix is used to linearize the system dynamics around the current state. The resulting predictions set the foundation for the subsequent update step, where real measurements are assimilated to refine the state estimate iteratively. This iterative process enhances the accuracy of the estimation as the filter adapts to changing system conditions over time.

- Correction step:

In the correction step of the EKF, real measurements are utilized to enhance the accuracy of the filter’s state estimate. The process begins by predicting expected sensor measurements based on the previously estimated state, incorporating a measurement model that may involve a nonlinear measurement function. The innovation, representing the difference between actual and predicted measurements, is then calculated. The Kalman gain, determined by the covariance matrices of the predicted state and measurements, as well as their cross-covariance, governs the weight assigned to the predicted state and the actual measurements during the update.

The state estimate is refined by adjusting it with the Kalman gain and the innovation. Simultaneously, the covariance matrix associated with the state is updated to reflect the reduced uncertainty after assimilating the measurements. This iterative process of prediction and correction optimally adapts the filter to dynamic system conditions, ensuring an accurate and continually refined estimation of the true system state.

According to the alteration method, $z_{k}$, which is the observation value, can be expressed as

(7) zk=Xk−x12+Yk−y12Xk−x22+Yk−y22Xk−x32+Yk−y32Xk−x42+Yk−y42+E1E2E3E4+δ1δ2δ3δ4

The observation vector comprises the distances between anchors and the mobile node, in addition to the observation noise. It is necessary to transform this vector into a linear approach.

The projection matrix H can be expressed as the Jacobian matrix of partial derivatives of $f\left(x,y\right)$ with respect to estimate state $x_{k}$, that is,

H=∂f1(Xk,Yk)∂Xk∂f1(Xk,Yk)∂X^k∂f1(Xk,Yk)∂Yk∂f1(Xk,Yk)∂Y^k∂f2(Xk,Yk)∂Xk∂f1(Xk,Yk)∂X^k∂f1(Xk,Yk)∂Yk∂f1(Xk,Yk)∂Y^k∂f3(Xk,Yk)∂Xk∂f1(Xk,Yk)∂X^k∂f1(Xk,Yk)∂Yk∂f1(Xk,Yk)∂Y^k∂f4(Xk,Yk)∂Xk∂f1(Xk,Yk)∂X^k∂f1(Xk,Yk)∂Yk∂f1(Xk,Yk)∂Y^k.

(8) $$=\left(\begin{array}{cccc} \frac{X_{k}-x_{1}}{\sqrt{{\left(X_{k}-x_{1}\right)}^{2}+{\left(Y_{k}-y_{1}\right)}^{2}}} & 0 & \frac{Y_{k}-y_{1}}{\sqrt{{\left(X_{k}-x_{1}\right)}^{2}+{\left(Y_{k}-y_{1}\right)}^{2}}} & 0\\ \frac{X_{k}-x_{2}}{\sqrt{{\left(X_{k}-x_{2}\right)}^{2}+{\left(Y_{k}-y_{2}\right)}^{2}}} & 0 & \frac{Y_{k}-y_{2}}{\sqrt{{\left(X_{k}-x_{2}\right)}^{2}+{\left(Y_{k}-y_{2}\right)}^{2}}} & 0\\ \frac{X_{k}-x_{3}}{\sqrt{{\left(X_{k}-x_{3}\right)}^{2}+{\left(Y_{k}-y_{3}\right)}^{2}}} & 0 & \frac{Y_{k}-y_{2}}{\sqrt{{\left(X_{k}-x_{3}\right)}^{2}+{\left(Y_{k}-y_{3}\right)}^{2}}} & 0\\ \frac{X_{k}-x_{4}}{\sqrt{{\left(X_{k}-x_{4}\right)}^{2}+{\left(Y_{k}-y_{4}\right)}^{2}}} & 0 & \frac{Y_{k}-y_{4}}{\sqrt{{\left(X_{k}-x_{4}\right)}^{2}+{\left(Y_{k}-y_{4}\right)}^{2}}} & 0 \end{array}\right).$$

Accordingly, the Kalman gain $K_{k}$ can be calculated in accordance with the following equation:

(9) $$K_{k}=P_{k}^{-}H^{T}{\left({HP}_{k}^{-}H^{T}+R_{k}\right)}^{-1}.$$

H is the matrix, which is a projection to turn $x_{k}$ into a position. $R_{k}$ is the measurement covariance matrix, and $e_{m}$ is the measurement noise vector, where each element follows the Gaussian distribution. The final position is ${\hat{x}}_{k}$, which can be calculated according to

(10) $${\hat{x}}_{k}=x_{k}^{-}+K_{k}\left(z_{k}-E\left(v_{k}\right)-f\left(x_{k}^{-}\right)\right).$$

This formular of ($z_{k}-E\left(v_{k}\right))$ is the observation value, and $E\left(v_{k}\right)={\left(E_{1},\;E_{2},E_{3}\right)}^{T}$ is the average range vector of anchors.

#### 3.5.2. Low-Pass Filter and Average Filter (LPF-AF)

The low-pass filter and average filter (LPF-AF) is frequently employed in robot localization to mitigate the effects of sensor noise and enhance the clarity of the data [21,31]. The low-pass filter algorithm permits the transmission of low-frequency signals while attenuating high-frequency noise, thereby facilitating the maintenance of stable and accurate position estimates by filtering out rapid, unnecessary fluctuations in the data. In contrast, the moving average filter algorithm employs a weighted average of a fixed number of consecutive measurements to achieve a smoother output. This approach effectively reduces random variations and noise in the data. By continuously averaging recent data points, the moving average filter provides a more consistent estimate of the robot’s position, thereby reducing the impact of outliers or sudden changes in sensor readings. The application of both filters contributes to an improvement in the reliability of the robot’s localization as a consequence of an enhancement in the quality of the sensor data. Algorithm 1 illustrates the methodology employed in the implementation of low-pass and moving average filters.
**Algorithm 1:** LPF-AF1:Input: data = ${[(x}_{raw}$, $y_{raw)},\;(x_{truth},\;y_{truth})]$2:Integrate LPF and AF3:Filtered_data = []4:  For i in range (len(data));5:    Initialize parameters for both AF and LPF:6:

 Choose a window size (N) for AF: N=57:


 N=5
8:

 Choose a smoothing constant (α) for the LPF9:


 Where 0<α<1 (α=0.2)10:

 Apply AF();11:


 For each time step t:12:


 Update the sum from nearby data points 13:


 Calculate average value from data points14:


 If t≥N, subtract the value that is leaving the window (i.e., the value from N step ago):15:


 Compute the AF output:16:


  x-coordinates and y-coordinates from AF17:

 Apply LPF on the AF output ();18:


 For each time step t≥0:19:


 Update the filtered x-coordinate by LPF-AF20:


 Update the filtered y-coordinate by LPF-AF21:  End for22:Output the final filtered by LPF-AF23:Filtered x-coordinate24:Filtered y-coordinate

LPF-AF is an algorithm used to smooth x- and y-coordinate data, reducing noise while maintaining the overall trend. The LPF uses a smoothing factor α (between 0 and 1) to calculate the filtered coordinates, blending the current coordinates with the previous filtered values. A smaller α offers greater smoothing but slower response, while a larger α responds faster to changes but reduces noise less effectively. In contrast, the AF averages the last N data points for both x- and y-coordinates, requiring a buffer of past values. A larger window size N produces smoother results but may delay the response. While the LPF is more suitable for real-time systems due to its simplicity, the AF provides straightforward averaging with potentially better noise reduction, depending on the chosen window size.

The LPF-AF algorithm combines the benefits of the LPF and AF to smooth x- and y-coordinate data. The LPF reduces noise by blending current coordinates with previous filtered values, offering a balance between a fast response and effective noise reduction. The AF averages the last N data points, providing further noise reduction but with a potential delay. By integrating both, LPF-AF achieves enhanced noise reduction while maintaining real-time performance, making it ideal for systems that require both accuracy and timely feedback.

#### 3.5.3. Low-Pass Filter and Kalman Filter (LPF-KF)

The combination of a low-pass filter and a Kalman filter (LPF-KF) represents a robust methodology for robot localization, with the objective of improving the precision and reliability of position estimates [21,31]. The low-pass filter is employed to attenuate high-frequency noise in sensor data, enabling the transmission of only the low-frequency, more pertinent information, which serves to mitigate the impact of abrupt, erratic fluctuations in the measurements. In contrast, the Kalman filter represents a more sophisticated algorithmic approach. It employs a dynamic model to predict the robot’s position and then updates this prediction by incorporating new sensor data while accounting for their inherent uncertainties. The integration of the LPF for the preprocessing of sensor data and the Kalman filter for real-time prediction and correction allows for an effective balance between noise reduction and accurate state estimation, thereby facilitating more reliable and precise localization of the robot. Algorithm 2 addresses the methodology for implementing the low-pass filter and Kalman filter (LPF-KF) combination. The process of using the low-pass filter to reduce high-frequency noise and the Kalman filter to refine position estimates through predictive modeling and updates is outlined. This enhances the accuracy and reliability of robot localization.
**Algorithm 2:** LPF-KF1:Input: data = ${[(x}_{raw}$,$\;y_{raw)},\;(x_{truth},\;y_{truth})]$2:Integrate LPF and KF3:Filtered_data = []4:  For i in range (len(data));5:    Initialize parameters for both KF and LPF:6:
 Initialize state vectors for x- and y-coordinates:7:
 Initialize state covariance matrices:8:
 Choose process noise covariance Q9:
 Choose a smoothing constant (α) for the LPF10:

 Where 0<α<1 (α=0.2)11:

 Apply LPF();12:


 For each time step t:13:


 Update the LPF filtered values for both x- and y-coordinates14:

 Apply KF();15:


 Process Noise:16:



 ${\hat{x}}_{k}=x_{k}+K_{k}\left(z_{k}-Hx_{k}\right)$
17:


 Correction Step:18:



 ${\;z}_{k}=H\;x_{k}+r_{k}$
19:


 Predict Step:20:


 Predict the next state based on the current state estimate21:



 $x_{k+1}=Ax_{k}+q_{k}$
22:


 Update Step:23:



 Use the LPF output as the measurement to correct the predicted state24:



 Kalman gain calculation25:


 Extract filtered coordinates:26:


 Update the filtered x-coordinate by LPF-KF27:


 Update the filtered y-coordinate by LPF-KF28:  End for29:Output the final filtered by LPF-KF30:Filtered x-coordinate31:Filtered y-coordinate

The LPF and KF are two techniques used for smoothing x- and y-coordinate data, focusing on noise reduction while preserving the underlying signal. The LPF works by applying a smoothing factor α that weights the current coordinates with the previous filtered values, blending them to reduce rapid fluctuations. A smaller α value results in greater smoothing, producing a slower response to changes, while a larger α allows the filter to adapt more quickly, with less noise suppression. This simplicity makes the LPF straightforward to implement, but it does not adapt to variations in noise levels over time. In contrast, the KF is a more sophisticated method that combines prediction and measurement updates to estimate the position of the coordinates. It starts by predicting the next state using a mathematical model, then corrects that prediction using new measurements. The degree of correction is determined by the Kalman gain, which is adjusted dynamically based on the uncertainties in the model and sensor measurements. This adaptability allows the KF to handle varying levels of noise more effectively, providing optimal estimates even in uncertain conditions, making it more suitable for complex scenarios compared to the simpler, fixed approach of the LPF.

Integrating the LPF with the KF combines their strengths, offering both simplicity and adaptability. The LPF smooths high-frequency noise initially, stabilizing the data for the KF to refine. The KF then adjusts predictions based on dynamic noise levels, providing more accurate and adaptable estimates. This hybrid approach enhances data smoothing and position estimation, improving responsiveness to varying noise and ensuring more reliable outputs.

#### 3.5.4. Low-Pass Filter and Extended Kalman Filter (LPF-EKF)

Algorithm 3 provides a comprehensive explanation of the methodology employed in the implementation of the “low-pass filter and extended Kalman filter (LPF-EKF)” approach [21,31]. This algorithm details the implementation of a low-pass filter to preprocess sensor data, thereby attenuating high-frequency noise, which is of paramount importance for the enhancement of data quality prior to its introduction into the EKF. Subsequently, the EKF performs advanced state estimation, accounting for nonlinearities in the system dynamics by predicting the robot’s position and correcting it based on the filtered sensor inputs. The combination of these two techniques is illustrated in detail in Algorithm 3. It demonstrates how the low-pass filter enhances the robustness and accuracy of the EKF, thereby improving the reliability of robot localization in complex, dynamic environments.
**Algorithm 3:** LPF-EKF1:Input: data = ${[(x}_{raw}$,$\;y_{raw)},\;(x_{truth},\;y_{truth})]$2:Integrate LPF and EKF3:Filtered_data = []4:  For i in range (len(data));5:    Initialize parameters for both EKF and LPF:6:
 Initialize state vectors for x- and y-coordinates:7:
 Initialize state covariance matrices:8:
 Choose process noise covariance Q9:
 Choose a smoothing constant (α) for the LPF10:

 Where 0<α<1 (α=0.2)11:

 Apply LPF();12:


 For each time step t:13:


 Update the LPF filtered values for both x- and y-coordinates14:

 Apply EKF();15:


 Process Noise:16:



 $z_{t}=C_{t}x_{t}+W_{t},W_{t}\sim Ɲ\left(0,Q_{t}\right)$
17:


 Correction Step:18:



 $z_{k}=H\;x_{k}+r_{k}$
19:


 Predict Step:20:


 Predict the next state based on the current state estimate21:



 $x_{t}=g\left(x_{t-1},\;u_{t},\;v_{t-1}\right)$
22:



 $z_{t}=h\left(x_{t},w_{t}\right).$
23:


 Calculate the Jacobian F of the state transition function24:



 Linearize the nonlinear function25:


 Update Step:26:



 Use the LPF output as the measurement to correct the predicted state27:



 Calculate the Jacobian F of the measurement function at the current predicted state28:



 Kalman gain calculation29:


 Extract filtered coordinates:30:


 Update the filtered x-coordinate by LPF-EKF31:


 Update the filtered y-coordinate by LPF-EKF32:  End for33:Output the final filtered by LPF-EKF34:Filtered x-coordinate35:Filtered y-coordinate

The utilization of a low-pass filter within an EKF for the purpose of robot localization presents several advantages, primarily in terms of enhanced accuracy and the stability of position estimates. The low-pass filter is an effective means of reducing high-frequency noise in sensor data, which can otherwise result in erratic or unreliable readings. By attenuating the noise before the data are processed by the EKF, the filter ensures that the EKF receives a more uniform and reliable input, which in turn allows for more precise predictions and corrections in the robot’s position. This preprocessing step assists the EKF in focusing on significant, low-frequency trends in the data, thereby enhancing its capacity to track the robot’s true state even in the presence of noisy or fluctuating measurements. Consequently, the combined use of the low-pass filter within an EKF results in more robust localization, particularly in environments with high levels of sensor noise or when dealing with complex, nonlinear system dynamics.

Integrating the LPF and EKF provides a robust approach for smoothing and estimating x- and y-coordinates. The LPF first reduces high-frequency noise by applying an exponential smoothing technique, which helps stabilize the data with a smoothing factor α. This simple filtering method effectively removes quick fluctuations but may not fully account for varying levels of noise. Once the data is smoothed, the EKF takes over by predicting the state using a model, then adjusting the predictions based on noisy measurements. The EKF uses the Kalman gain to dynamically correct the estimates, providing an optimal balance between the model and measurements, especially in nonlinear systems. This combination leverages the LPF’s ability to reduce noise quickly and the EKF’s strength in adapting to changes in noise and uncertainties, leading to more accurate and reliable position estimates in dynamic environments.

#### 3.5.5. Neural Network-Based EKF (NN-EKF)

Algorithm 4 combines an extended Kalman filter (EKF) with a neural network model to process and predict data. The process begins with input data, which consists of raw and ground truth values. These data are then processed by the EKF to filter and prepare the dataset for further analysis. The filtered data are then fed through a neural network model, specifically a Multi-layer Perceptron (MLP) Regressor. The objective of this MLP is to enhance predictive accuracy by identifying the relationships between the filtered inputs and their corresponding outputs.
**Algorithm 4:** NN-EKF1:Input: data = ${[(x}_{raw}$,$\;y_{raw)},\;(x_{truth},\;y_{truth})]$2:Loading dataset processed by EKF3:Data normalization: Minimum and Maximum Scaler (MinMaxScaler)4:NN-EKF (filtered data)5:Neural network model setup6: Multi-layer Perceptron Regressor (MLP)7:
Input layer:8:
Two neural network regressors (net-x and net-y) are created to predict the $x_{i}$ and $\;y_{i}$ positions.9:
Hidden layer:10:
Single hidden layer with 50 neurons11:

Parameters:12:

Initialize weights and biases $w_{i}$:13:

Activation function: ReLU14:

Solver: Adam optimizer for efficient training15:

Alpha: 0.001, regularization parameter to avoid overfitting16:

Max iterations: 2000 iterations17:

Learning rate: 0.1, controlling the step size during training18:

Training process for optimizing loss function 19:

For episode in range (500):20:

 Data splitting (60% for training and 40% for testing)21:


Neural networks are trained on the training set for both x- and y-coordinates22:


Total loss = 023:


For i in range(lens(data)):24:


  Calculate loss: Mean Squared Error (MSE)25:



MSE = $\frac{1}{n}\sum_{1}^{n}{\left(x_{i}-y_{i}\right)}^{2}$26:



Backpropagation and weight update 27:



Update weights and biases28:


End for29:


Output layer:30:


Inverse transformation from normalization data31:


Optimal position filtering32:

End for33:
Print (filtered data)34:
Output: data = $\left(x_{predict},\;y_{predict}\right)$35:
Euclidean distance loss

The neural network model, which forms the basis of the algorithm of the neural network-based EKF (NN-EKF), comprises three layers: an input layer, a single hidden layer, and an output layer [16]. The input layer receives the state values, which include both the unfiltered and filtered (estimated) x- and y-coordinates. The network is initialized with weights (w) and biases (b), which are then adjusted during the training process in order to achieve the best possible results. The hidden layer employs the ReLU (Rectified Linear Unit) activation function, which introduces nonlinearity into the model, enabling it to capture more complex patterns in the data. The core of the algorithm lies in the training process, where the network aims to minimize the prediction error by optimizing a loss function, specifically the mean squared error (MSE).

During the training phase, the algorithm employs backpropagation to compute gradients, which directs the updating of weights and biases in conjunction with the Adam optimizer. This process is repeated until the total loss across multiple epochs is reduced to a satisfactory level, at which point the model is deemed to have achieved an acceptable level of performance. Once the training phase is complete, the final output layer produces the predicted values for the input data. This results in a set of predictions (x_predict, y_predict) that the model has learned to generate based on the training it received. The algorithm generates these predictions, which can be utilized for further analysis or decision-making processes.

Combining the EKF with a neural network can significantly improve indoor localization, ensuring precise and reliable robot movements. The EKF is effective at fusing noisy sensor data, such as odometry or distance sensors, to estimate the robot’s position. When combined with a neural network, which can learn complex patterns and dynamics, it further enhances the precision of state estimation, especially by handling nonlinearities more effectively than an EKF alone. Additionally, the neural network helps model and predict sensor noise patterns, allowing the EKF to better filter out erroneous data, leading to more accurate position estimates. The neural network can also learn from historical movement data, improving the EKF’s ability to predict future states, particularly in complex indoor environments with dynamic obstacles. This combination also enables adaptive filtering, as the EKF can refine its process based on the neural network’s insights, ensuring better localization under changing conditions. Neural networks optimize sensor fusion, providing a more accurate overall estimate by correlating multiple sensor inputs, and are especially useful for handling nonlinear relationships in robot kinematics or sensor inaccuracies. The integration improves robustness, allowing the system to generalize well in diverse and unpredictable indoor settings. Finally, with well-optimized neural networks, the combined system provides real-time updates to the EKF, ensuring quick adaptation and accurate localization for robot movement. This synergy enhances both the precision and reliability of indoor localization systems. While the NN-EKF combination offers benefits in specific scenarios, DNNs are generally more scalable, adaptable, and efficient at handling complex, nonlinear data, making them a better choice for large-scale or highly dynamic indoor localization tasks. So DNN-EKF is best for handling complex, nonlinear data, which is taken as our proposed approach in this work.

#### 3.5.6. Deep Neural Network-Based Extend Kalman Filter (DNN-EKF)

In algorithm 5, the DNN-EKF fuses a deep neural network (DNN) with an extended Kalman filter (EKF) to elevate prediction precision in a series of steps. The input dataset comprises raw state measurements and their corresponding true values. At the outset, the algorithm loads a dataset that has been preprocessed by the EKF. This step is of great importance, as the EKF assists in reducing noise and filtering out inaccuracies from the raw data, thus making it more suitable for training the neural network. The filtered data are then prepared for use in the neural network, where the core processing will take place. At the core of the algorithm is the Multi-layer Perceptron (MLP) Regressor, a type of neural network that performs regression tasks. The MLP is structured into three main layers: the input layer, the hidden layers, and the output layer. The input layer receives the state values, including both the unfiltered and EKF-filtered measurements. The network’s neurons are initialized with weights and biases, and the ReLU (Rectified Linear Unit) activation function is applied in the hidden layers to introduce nonlinearity, allowing the network to capture complex patterns in the data. The network’s architecture begins with a single hidden layer, but additional hidden layers can be incorporated for more in-depth learning.

As data traverse the network, each subsequent layer processes the input from the previous layer, applying weights, biases, and activation functions in an iterative manner. This multi-layer approach allows the network to model complex relationships between the input features and the target outputs. Subsequently, the algorithm enters a training phase, during which the loss function is optimized across multiple epochs. The mean squared error (MSE) is used as the loss function, which quantifies the discrepancy between the predicted and actual values. The backpropagation algorithm is used to adjust the weights and biases in order to minimize the loss, with the Adam optimizer facilitating efficient and adaptive updates during training.

The final stage of the process is the generation of the predicted state values, which is achieved by combining the activations from the last hidden layer. The algorithm then generates the predicted data, which includes the predicted state values (x_predict, y_predict). The EKF-enhanced data stream plays a pivotal role in optimizing the neural network’s performance, ensuring more accurate and reliable inputs, which in turn deliver superior predictions. The result is a robust DNN-EKF model that effectively integrates traditional filtering techniques with modern deep learning methods, resulting in improved prediction accuracy.
**Algorithm 5:** DNN-EKF1:Input: data = ${[(x}_{raw}$,$\;y_{raw)},\;(x_{truth},\;y_{truth})]$2:Loading dataset processed by EKF3:Data normalization: Minimum and Maximum Scaler (MinMaxScaler)4:DNN-EKF (filtered data)5:Neural network model setup6:  Multi-layer Perceptron Regressor (MLP)7:
Input layer:8:
Two neural network regressors (net-x and net-y) are created to predict the $x_{i}$ and $\;y_{i}$ positions.9:
Hidden layer:10:
3 hidden layers with 50 neurons for each layer11:

Parameters:12:

Initialize weights and biases $w_{i}$:13:

Activation function: ReLU14:

Solver: Adam optimizer for efficient training15:

Alpha: 0.001, regularization parameter to avoid overfitting16:

Max iterations: 2000 iterations17:

Learning rate: 0.1, controlling the step size during training18:

Training process for optimizing loss function 19:

For episode in range (500):20:

 Data splitting (60% for training and 40% for testing)21:


Neural networks are trained on the training set for both x- and y-coordinates22:


Total loss = 023:


For i in range(lens(data)):24:


  Calculate loss: Mean Squared Error (MSE)25:



MSE = $\frac{1}{n}\sum_{1}^{n}{\left(x_{i}-y_{i}\right)}^{2}$26:



Backpropagation and weight update 27:



Update weights and biases28:


End for29:


Output layer:30:


Inverse transformation from normalization data31:


Optimal position filtering32:

End for33:
Print (filtered data)34:
Output: data = $\left(x_{predict},\;y_{predict}\right)$35:
Euclidean distance loss

The combined EKF and DNN model utilizes three hidden layers, each with 50 neurons, while the neural network version uses only one hidden layer with 50 neurons. The addition of extra hidden layers in the DNN enables it to learn more complex, hierarchical patterns in the data, enhancing its ability to model nonlinear relationships and capture more detailed feature representations from the sensor inputs. Each hidden layer in the DNN allows the model to build progressively more abstract representations of the data, improving its ability to understand complex correlations between different sensor readings and the robot’s position. In contrast, the simpler neural network with a single hidden layer can only model relatively shallow and basic relationships, making it less capable of handling intricate or highly nonlinear patterns in the data.

With three hidden layers, the DNN can learn from more layers of abstraction, making it better suited to handle dynamic, nonlinear, and complex environments commonly encountered in indoor localization. This deeper architecture allows the DNN to generalize better across different environments, enabling it to process a variety of sensor inputs more effectively and adapt to new or unseen situations. Additionally, the extra layers enhance the network’s robustness by allowing it to better handle noisy or incomplete data, improving the overall accuracy of state estimation. The benefit of the deeper architecture in the DNN is that it provides richer and more robust feature extraction capabilities, allowing the model to learn more intricate dependencies between sensor data and the robot’s position. This enables the DNN to overcome the limitations of a simpler neural network by improving its predictive performance, leading to more precise, reliable, and adaptive localization. The increased depth helps the DNN capture long-term dependencies and subtle patterns in the data, which a shallow neural network might miss, ultimately improving robot movement in dynamic, cluttered, or complex indoor environments.

### 3.6. Proposed System Flow Diagram

**Figure 4:**
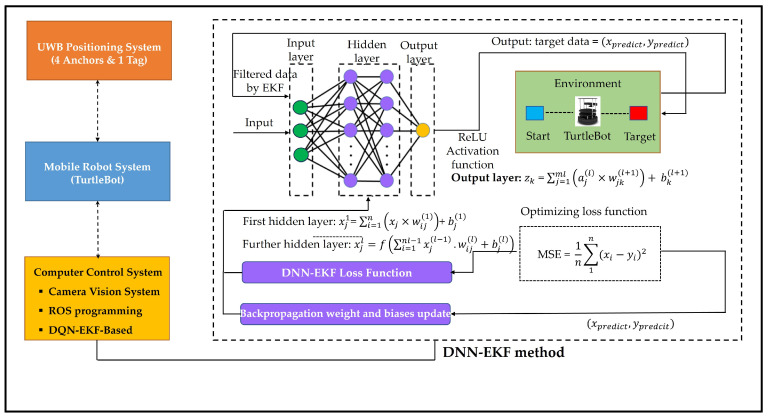
Proposed DNN-EKF flow diagram.

Figure 4 shows the process in our proposed system flow diagram of DNN-EKF, which begins with the acquisition of a dataset containing input features and target labels, followed by data preprocessing to handle missing values and outliers. The DNN-EKF architecture is defined, including the configuration of layers, selection of activation functions, and specifications for the output layer. The DNN typically consists of an input layer, several hidden layers, and an output layer, each with a specific number of neurons depending on the complexity of the problem. The hidden layers are of critical importance to the DNN’s capacity to discern intricate patterns within the data. Each hidden layer is comprised of multiple neurons, wherein each neuron performs a weighted sum of its inputs, adds a bias, and then applies an activation function. We utilized the Rectified Linear Unit (ReLU) activation function, which is a good choice due to its ability to introduce nonlinearity into the model while maintaining computational efficiency. ReLU activation functions can enable the network to learn complex representations of the data. The depth and number of neurons in these hidden layers play a pivotal role in determining the model’s capacity to learn intricate relationships between inputs and outputs.

The process of training the DNN entails the minimization of the discrepancy between the predicted and actual target values, a procedure that is governed by a loss function. The loss function of the mean squared error (MSE) for regression tasks can provide a good correspondence between the model’s predictions and the actual outcomes. The objective during the training phase is to adjust the network’s weights and biases in order to minimize the loss function, thereby enhancing the accuracy of the model. This optimization is conducted through the use of an algorithmic technique known as backpropagation, which calculates the gradient of the loss function with respect to each parameter within the network. The gradients indicate the direction and magnitude of the requisite adjustments to the weights and biases to reduce the loss.

Backpropagation is comprised of two principal stages: the forward pass and the backward pass. During the forward pass, the input data flows through the network, with each neuron in the hidden layers applying its activation function (e.g., ReLU) to produce the output. Subsequently, the output is compared with the target values, and the loss is computed. In the backward pass, the algorithm calculates the gradients of the loss with respect to each weight and bias, proceeding from the output layer in a backward direction to the input layer. Subsequently, the gradients are employed to update the weights and biases, typically using the Adam optimization technique.

This iterative process of adjusting parameters continues until the loss function has reached an acceptable level of minimization. Validation is conducted on a separate dataset to fine-tune hyperparameters and prevent overfitting. Testing, on the other hand, evaluates the model’s performance on unseen data, and it is used to inform the final deployment of the trained model for real-world predictions.

## 4. Experiment Result and Analysis

To ensure the effective implementation of the DNN-EKF algorithm, it is essential to use the traditional EKF model integrated with the DNN. The goal of this integration is to increase the capacity of the model and improve the localization accuracy for mobile robots, enabling more reliable and precise navigation in dynamic environments. By leveraging the complementary strengths of EKF’s simplicity and DNN’s powerful feature extraction capabilities, the proposed hybrid approach aims to overcome the limitations of each model when used independently.

The following section provides a detailed explanation and analysis of the evaluation metrics, the proposed method, and a comprehensive review of existing work on localization methods. This discussion includes a thorough examination of the techniques and algorithms used in related research, highlighting their performance, adaptability, and applicability to different scenarios. The goal of this section is to provide a comprehensive understanding of the methodologies employed and to provide insight into the strengths and limitations of the proposed approach in comparison to existing work. It also aims to identify potential areas for improvement, guide future research directions, and demonstrate the practical implications of the proposed method for real-world mobile robot applications.

### 4.1. Experimentation Environment

In our research, we employ sophisticated simulation tools, namely ROS (Robot Operating System) programming and Python with the scikit-learn library, to facilitate the advancement of robotic system development and analysis. ROS (Robot Operating System) programming provides a structured and modular framework that facilitates the seamless communication and coordination of various components within a robotic system. It permits the development of sophisticated algorithms, the integration of sensors, and the implementation of efficient control mechanisms, which are essential for enabling autonomous decision-making and adaptive behaviors in robots. The modularity of the ROS also ensures that the system can be readily extended or modified, thus facilitating the iterative development and testing processes that are essential in robotics research. Also, we utilize RViz (ROS Visualization), which is a powerful 3D visualization tool for the ROS (Robot Operating System) that allows you to visualize robots, sensor data, and the state of an ROS-enabled environment. It is commonly used in mobile robot simulation to visualize the robot’s movements, sensor data, and the environment in real time. We determined a step-by-step process of using RViz for mobile robot simulation.

Furthermore, the integration of Python with the scikit-learn library enhances the adaptability of our methodology. The scikit-learn library provides comprehensive tools for machine learning and data analysis, including capabilities for model training, predictive analysis, and data processing. The library supports a wide range of algorithms, from simple linear models to complex ensemble methods, which are invaluable for tasks such as classification, regression, clustering, and dimensionality reduction. The incorporation of these tools enables the expeditious prototyping and validation of models, thereby facilitating the investigation of diverse methodologies to enhance the accuracy and efficiency of robotic systems.

The combination of ROS programming and Python with scikit-learn facilitates the simulation of complex scenarios, the analysis of system behavior, and the optimization of robotic system performance in an efficient manner. By employing the ROS to simulate authentic, real-world environments and Python to process and interpret the resulting data, we can conduct iterative refinements to our algorithms and models, thereby enhancing their capacity to cope with the challenges posed by dynamic and unpredictable settings. This comprehensive integration serves to underscore the significance of combining the ROS and machine learning tools in order to drive advancements in robotic research and development. This demonstrates the potential for these technologies to work in concert, facilitating the development of more intelligent, adaptive, and capable robotic systems that can operate effectively in a diverse range of applications, from autonomous navigation to complex task automation. This approach not only accelerates the development cycle but also paves the way for more innovative solutions in the field of robotics.

### 4.2. Performance Metrics for Result Evaluation

In this paper, we utilize a set of comprehensive performance metrics to rigorously evaluate the proposed methodology’s effectiveness in robot localization. The primary metrics include robot localization accuracy measured along the x- and y-coordinate values, providing insights into the precision of the system in determining the robot’s position within its environment. Additionally, we assess the average distance loss, expressed in millimeters (mm), as a crucial measure of the discrepancy between predicted and actual robot positions. This metric offers valuable information about the overall accuracy of the proposed methodology across various spatial coordinates.

To further quantify the localization performance, we utilize the mean squared error (MSE), a statistical measure that gauges the average squared difference between predicted and actual values. MSE is a metric that measures the accuracy of predictions by comparing them to actual values. In robot localization, it evaluates how closely the predicted positions match the robot’s true positions. MSE is calculated by taking the difference between predicted and actual coordinates, squaring these differences (to ensure they are positive), and averaging them. This is done separately for the x- and y-coordinates, giving insight into the accuracy in each dimension. A lower MSE indicates predictions are close to the true positions, showing good model performance, while a higher MSE points to larger prediction errors, indicating potential areas for improvement. This metric is particularly useful for penalizing larger errors more heavily, thus offering a clear indication of significant deviations in the robot’s predicted location. Additionally, we consider the Root Mean Squared Error (RMSE), which provides a more interpretable measure by returning the error in the same units as the original data, thereby offering an intuitive understanding of the error magnitude.

We also incorporate the evaluation of localization robustness by analyzing the system’s performance under varying levels of noise and environmental changes. This includes assessing the system’s ability to maintain accurate localization in the presence of sensor inaccuracies, dynamic obstacles, and other real-world challenges. The robustness metrics are crucial for understanding how well the proposed methodology generalizes across different scenarios and how resilient it is to variations in the operating environment.

Finally, we conduct a comparative analysis with existing localization techniques by examining these metrics across a range of standard benchmarks. This comparison helps to position the proposed methodology within the broader context of robot localization research, highlighting its competitive advantages as well as potential areas for refinement. These performance metrics collectively contribute to a comprehensive and nuanced evaluation, allowing for a detailed analysis of the proposed methodology’s strengths and areas for improvement in the realm of robot localization.

### 4.3. Experiments and Parameter Settings

The experimental setup is conducted in two phases: the first phase involves real-world robot experimentation, while the second phase focuses on applying the collected dataset to the DNN-EKF model. In the first phase, the robot performs tasks in an indoor environment, gathering positional and sensor data that are critical for accurate localization. In the second phase, the collected data are preprocessed and normalized before being used to train and evaluate the DNN-EKF model. This dual-phase approach ensures that the model is tested on real-world data, making it more robust and capable of handling dynamic environments. By integrating both real-world experimentation and data-driven model training, the setup allows for a comprehensive evaluation of the model’s performance in practical scenarios.

The collected data are preprocessed and normalized after that using deep neural network models to predict and analyze positions based on the raw input data. The data are initially imported and normalized using the MinMaxScaler function to ensure that all features are on a common scale, which enhances the performance of the neural network. The dataset is preprocessed with MinMaxScaler before applying the model DNN-EKF. Preprocessing with MinMaxScaler is important because it normalizes the features of a dataset to a consistent scale, typically between 0 and 1. This is crucial for algorithms that are sensitive to the scale of input data, such as gradient descent-based and distance-based models. MinMaxScaler helps improve the convergence rate during training and ensures that no feature dominates the learning process due to its larger range. Two distinct neural network models are configured using the MLPRegressor class, with the objective of predicting the x- and y-coordinates, respectively. The DNN-EKF model uses three hidden layers with 50 neurons in each layer, while the NN-EKF model uses only one hidden layer with 50 neurons. This demonstrates that DNN-EKF can perform more effectively when working on complex tasks. We utilize ReLU as the activation function in the DNN-EKF model. ReLU enables the neural network to learn complex patterns in data, which are essential for accurate localization. In contrast to activation functions such as the sigmoid or tanh, ReLU does not saturate in the positive range, thereby avoiding the issue of vanishing gradients during backpropagation. This allows for more effective training of deeper networks, enabling them to capture complex relationships in robot motion. The output of the x- and y-coordinates in the localization process can span a wide range. ReLU is capable of handling outputs with a wide range, as it does not have an upper bound. This flexibility allows the network to learn without being constrained by the limitations of a bounded activation function, making it an ideal choice for continuous prediction tasks such as localization. In the context of robot localization, the precise prediction of x- and y-coordinates can be considered a regression problem. ReLU is an effective solution for regression tasks due to its linear and unbounded nature for positive inputs. Unlike other functions, it can produce a wide range of values, which is essential for accurate coordinate predictions without artificially limiting the output. The Adam optimizer, with a learning rate of 0.1 and a maximum of 2000 iterations, is selected for the optimized tuning parameters in the proposed model. The dataset is divided into a training set of 60% and a testing set of 40%, providing an effective balance between learning and evaluation. The 60% training set allows the model to learn sufficient patterns and relationships within the data, while the 40% testing set offers a robust evaluation sample to assess the model’s performance. This larger testing set improves the reliability of performance metrics, helps detect errors, and evaluates the model’s ability to generalize to new, unseen data. The split reduces the chance of overfitting, ensuring that the model performs well not just on the training data but also in real-world scenarios. To prevent overfitting, we utilize early stopping, a technique that halts the training process once the model achieves optimal results on the validation set. Early stopping helps prevent the model from continuing to train past the point where it might start to memorize the training data, thus ensuring better generalization to unseen data. The mean squared error (MSE) and Euclidean distance loss between the predicted and ground truth positions are calculated in order to assess the model’s performance.

The model’s performance is evaluated over the course of 500 episodes, with the episode exhibiting the minimum Euclidean distance loss being identified. For this episode, detailed plots are generated to visualize the predicted positions in comparison to the ground truth, the actual robot positions, and the results obtained through alternative methods. The code concludes by printing the episode number with the minimum loss, thereby providing insights into the optimal model configuration. The simulation parameters are summarized in Table 1.

### 4.4. Experiment Results

In this section, we will undertake a comprehensive analysis and comparison of the performance of our proposed model, examining several key metrics and their implications.

- Distance Loss Average: This metric provides a measure of the overall accuracy of our model in terms of how well it predicts distances. It quantifies the average deviation between the predicted and actual distances across all data points.
- Mean Squared Error (MSE) for x Position: The mean squared error (MSE) for the x position is a metric that assesses the precision of the model’s predictions along the x-coordinates. The MSE is calculated by averaging the squared differences between the predicted and actual x-coordinates.
- Mean Squared Error (MSE) for y Position: Similarly, the mean squared error for the y position is employed to assess the model’s accuracy in predicting the y-coordinates. By computing the mean squared error between the predicted and actual y positions, insights can be gained into the model’s performance in the vertical dimension.
- Trajectory Analysis: This entails a comparison of the predicted paths with the actual trajectories, with the objective of evaluating the degree of alignment between the model’s output and the actual movement patterns.

#### 4.4.1. Proposed Model with Learning Rate (lr) of 0.001 and Other Existing Works

**Figure 5:**
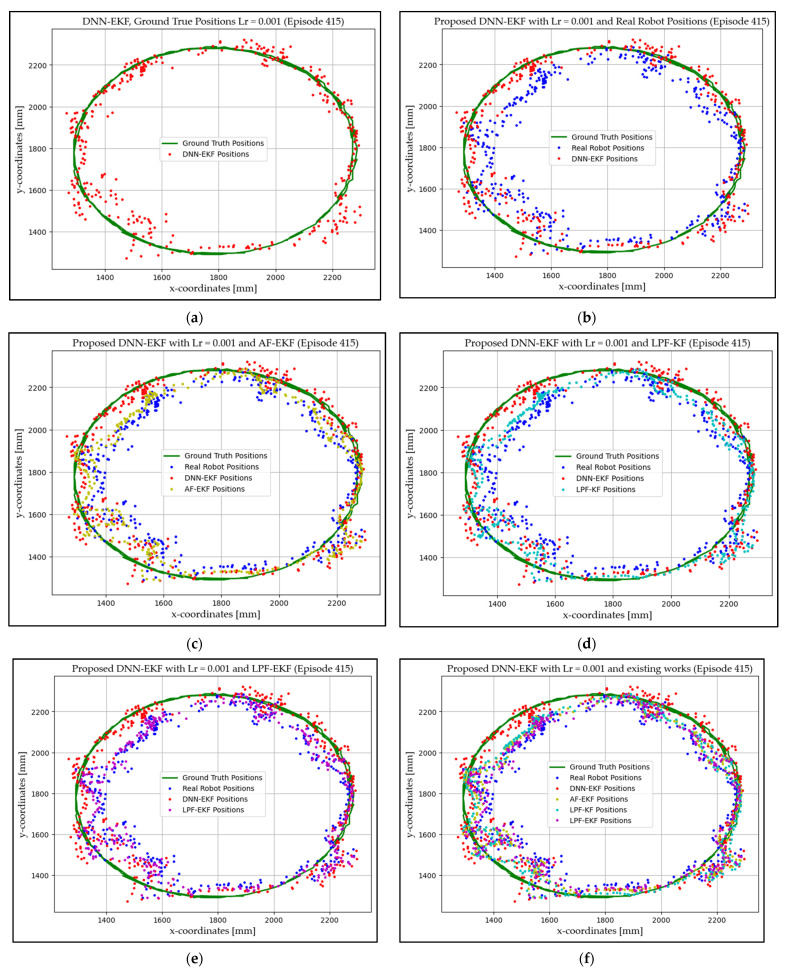
Proposed model with learning rate 0.001: model and ground truth positions (**a**), proposed model with real robot positions (**b**), proposed model and AF-EKF (**c**), proposed model and LPF-KF (**d**), proposed model and LPF-EKF (**e**), proposed model and all methods (**f**).

To demonstrate the enhanced performance of the proposed model DNN-EKF in comparison to alternative methodologies, it is instructive to examine the specifics of the outcomes illustrated in Figure 5. This figure presents a comprehensive array of contrasts across a range of metrics and visual representations for diverse models. The following is a detailed account of the insights offered by each subplot regarding the efficacy of the proposed model. Figure 5a depicts the trajectory predicted by the DNN-EKF model in relation to the ground truth positions. The accuracy of the predicted trajectory in relation to the ground truth indicates the extent to which the DNN-EKF model can follow the true path. A close alignment between the predicted and ground truth positions highlights the effectiveness of the model in capturing the true motion dynamics. Figure 5b depicts the proposed model with real robot positions. In this section, the predicted positions from the DNN-EKF model are compared with the actual positions recorded from a real robot. This visualization facilitates comprehension of the model’s performance in practical scenarios, as opposed to theoretical or simulated environments. The proximity of the predicted positions to the actual robot positions illustrates the model’s practical efficacy.

Next, Figure 5c illustrates the performance of the proposed model DNN-EKF and AF-EKF-based models. This subplot presents a comparison of the trajectories of the proposed model with those of the AF-EKF model. The trajectory of the proposed model should, in theory, exhibit a greater degree of correspondence with the ground truth or actual robot positions than that of the AF-EKF model, thereby underscoring its superior performance. This visual comparison facilitates the identification of the proposed model’s relative advantages in terms of accuracy and robustness. Figure 5d shows the proposed DNN-EKF and LPF-KF: this subplot presents a comparison of the performance of the proposed model with that of the LPF-KF model. An improved alignment of the proposed predictions with the ground truth or real positions, as compared to the LPF-KF model, would indicate that the former possesses superior predictive capabilities and reliability. Figure 5e depicts the performance of the DNN-EKF and LPF-EKF models.

This comparison demonstrates the relative performance of the proposed model in comparison to the LPF-EKF model. By examining this subplot, one can ascertain whether the proposed model DNN-EKF offers more precise and reliable predictions in comparison to the LPF-EKF model, thereby substantiating its efficacy. Finally, Figure 5f shows the proposed DNN-EKF model and all methods: the final subplot offers a comprehensive view by comparing the DNN-EKF model against all other methods (AF-EKF, LPF-KF, LPF-EKF). This holistic comparison allows for an overall assessment of the proposed model’s performance in relation to all the other methods tested. It is expected that the proposed model will show the best performance across all comparisons, as evidenced by the closest trajectory alignments with ground truth or real robot positions and minimized prediction errors.

A detailed comparison of the subplots reveals that the proposed DNN-EKF model consistently demonstrates superior performance. The predicted trajectories exhibit a high degree of fidelity to the ground truth or real robot positions, outperforming alternative methods such as AF-EKF, LPF-KF, and LPF-EKF in terms of accuracy and reliability. This robust performance can be attributed to the integration of deep neural networks with the extended Kalman filter, which enhances the model’s ability to accurately predict and adapt to complex motion dynamics.

#### 4.4.2. Proposed DNN-EKF with Learning Rate (lr) of 0.01, 0.1, and Other Existing Works

**Figure 6:**
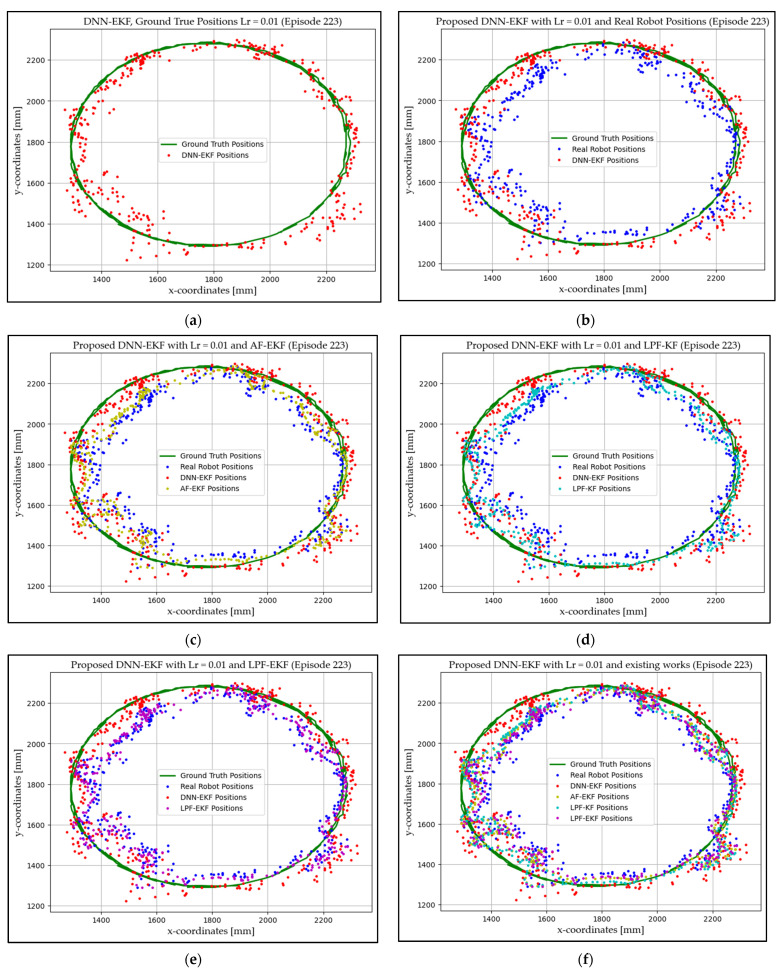
Proposed model with learning rate 0.01: model and ground truth positions (**a**), proposed model with real robot positions (**b**), proposed model and AF-EKF (**c**), proposed model and LPF-KF (**d**), proposed model and LPF-EKF (**e**), proposed model and all methods (**f**).

**Figure 7:**
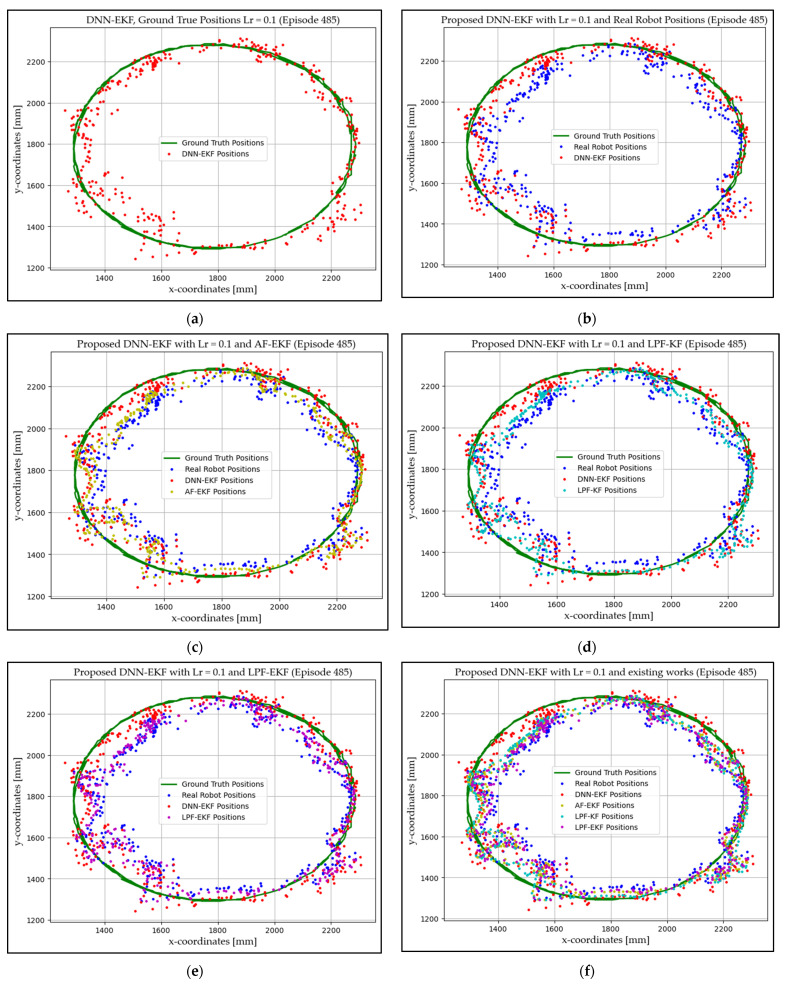
Proposed model with learning rate 0.1: model and ground truth positions (**a**), proposed model with real robot positions (**b**), proposed model and AF-EKF (**c**), proposed model and LPF-KF (**d**), proposed model and LPF-EKF (**e**), proposed model and all methods (**f**).

To further enhance the performance of our proposed DNN-EKF model, we conducted additional experiments, as depicted in Figure 6 and Figure 7. These figures explore the impact of various learning rates on the model’s performance, enabling us to fine-tune the model for optimal results.

The results presented in Figure 6a offer a detailed comparison between the DNN-EKF trajectories and ground truth trajectories, thereby providing crucial insights into the accuracy of the proposed methodology’s movement predictions. Figure 6b offers a detailed comparison between the raw robot trajectories based on UWB, the ground truth trajectories, and DNN-EKF, providing crucial insights into the accuracy of the proposed methodology’s movement predictions. The analysis includes a key metric, “distance loss”, revealing that, on average, the predicted trajectories deviate from the actual trajectories by approximately 76.73 mm. This metric serves as a comprehensive measure of the overall discrepancy between the target and actual positions of the robot, shedding light on the precision of the proposed methodology in determining the robot’s location. Furthermore, the MSE values for both the x- and y-coordinates provide a quantitative assessment of the accuracy. The calculated MSE for the x-coordinate is 64.56, while for the y-coordinate, it is 60.13. These MSE values represent the average squared differences between the robot’s positions and the ground truth along each coordinate, with lower values indicating a higher level of accuracy.

Next, the outcomes depicted in Figure 6c present an intricate juxtaposition of the original robot trajectories based on UWB, AF-EKF, DNN-EKF, and the ground truth trajectories, delivering crucial insights into the precision of the proposed methodology’s movement predictions. Within this analysis, a significant metric, denoted as “distance loss”, discloses that, on average, the predicted trajectories deviate from those derived through AF-EKF by approximately 76.20 mm. These metrics function as a comprehensive gauge of the overall disparity between the anticipated and actual positions of the robot, elucidating the accuracy of the proposed methodology in pinpointing the robot’s location. Moreover, the MSE values for both the x- and y-coordinates furnish a quantitative evaluation of accuracy. The computation MSE for the x-coordinate stands at 55.16, while for the y-coordinate, it registers at 66.26.

These MSE values represent the mean squared differences between the robot’s positions and the ground truth along each coordinate, with diminished values indicating a heightened level of accuracy.

Figure 7e is the existing work in reference [31], which is used as a comparing scheme with our proposed algorithm. It presents details and offers a comprehensive assessment of the performance metrics for the low-pass filtering integrated with the extended Kalman filter (LPF-EKF) methodology. The “distance loss” average of 75.30 mm signifies the average discrepancy between the predicted trajectories using LPF-EKF and the actual trajectories, providing a measure of how closely the predicted positions align with the ground truth. This metric is crucial in evaluating the accuracy and precision of the LPF-EKF methodology in determining the robot’s location, with a lower distance loss indicating a more accurate prediction. The MSE values provide a quantitative evaluation of the accuracy along each coordinate. For the x-coordinate, the computation MSE is 58.93, while for the y-coordinates, it is 59.44. The MSE represents the average squared differences between the predicted positions and the ground truth position along each respective coordinate.

A lower MSE indicates that the predicted positions are closer to the actual positions, highlighting the precision of the LPF-EKF methodology in minimizing errors along both the horizontal (x) and vertical (y) coordinates. These detailed metrics collectively contribute to a nuanced understanding of the LPF-EKF methodology’s strengths and areas for potential improvement in accurately localizing the robot within its environment.

**Figure 8:**
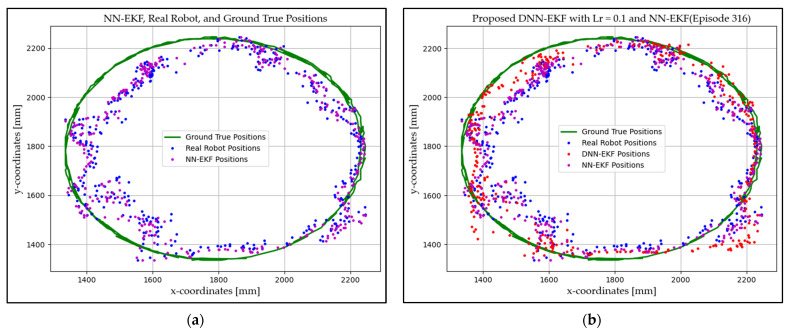
Proposed model with learning rate 0.1: NN-EKF, ground truth positions, and real robot positions (**a**), proposed model and NN-EKF, ground truth positions, and real robot positions (**b**).

Next, Figure 8 is the existing work in reference [16], which is also used as a second comparing scheme with our proposed algorithm. The provided details offer a thorough examination of the performance metrics for the extended Kalman filter with neural network (NN-EKF) methodology. The “distance loss” average of 73.35 mm represents the mean discrepancy between the predicted trajectories using NN-EKF and the actual trajectories, offering insight into how closely the predicted positions align with the ground truth. This metric serves as a key indicator of the accuracy and precision of the NN-EKF methodology in estimating the robot’s location. A lower distance loss suggests a more accurate prediction, highlighting the efficacy of the NN-EKF approach in localizing the robot within its environment. The MSE values provide a quantitative evaluation of accuracy along each coordinate. For the x-coordinate, the computation MSE is 55.16, while for the y-coordinate, it is 66.26. The MSE represents the average squared differences between the predicted positions and the ground truth positions along each respective coordinate. A lower MSE indicates a closer alignment between the predicted and actual positions, emphasizing the precision of the NN-EKF methodology in minimizing errors along both the horizontal (x) and vertical (y) coordinates. These detailed metrics collectively contribute to a nuanced understanding of the NN-EKF methodology’s performance, offering valuable insights into its strengths and areas for potential improvement in robotic localization applications.

Figure 8b shows the detailed results of the proposed DNN-EKF approach, with a learning rate set to 0.1, comparing it to the existing work in reference [16], which is NN-EKF. It offers valuable insights into the performance of the methodology in indoor localization as compared to ground truth positions, and real robot positions. The “distance loss” average, measuring approximately 68.06 mm, is a crucial metric that reflects the mean discrepancy between the predicted robot trajectories and the actual trajectories. This metric is essential for assessing the overall accuracy of the proposed methodology in predicting the robot’s movement within an indoor environment. A lower distance loss indicates a more accurate prediction, demonstrating the efficacy of the methodology in aligning predicted positions with the ground truth. The MSE values for both the horizontal (x) and vertical (y) coordinates further provide a quantitative measure of accuracy. The calculated MSE for the x-coordinate is 56.63, and for the y-coordinate, it is 56.82. These values represent the average squared differences between the predicted positions and the ground truth positions along each respective coordinate. A lower MSE suggests a higher level of accuracy in predicting positions, highlighting the precision of the proposed methodology. The combination of a low distance loss average and MSE values indicates that the proposed work, with a learning rate of 0.1, exhibits promise for achieving precise and reliable indoor localization. These results underscore the potential of the methodology for real-world applications in robotics, displaying its ability to accurately predict and localize the robot within an indoor environment. Table 2 shows all the results for both the horizontal (x) and vertical (y) coordinate MSE values, and we can see that the proposed method produces less error than other existing work.

Based on the detailed results obtained from the experiments, it is evident that the proposed methodology employing a learning rate of 0.1 outperforms other configurations. The distance loss average of approximately 68.06 mm displays the method’s effectiveness in predicting robot trajectories, indicating a smaller mean discrepancy compared to alternative learning rates. This lower distance loss underlines the enhanced accuracy of the proposed methodology, emphasizing its proficiency in aligning predicted positions with the actual trajectories.

#### 4.4.3. Distance Loss Based on Proposed DNN-EKF, NN-EKF, and LPF-EKF

**Figure 9:**
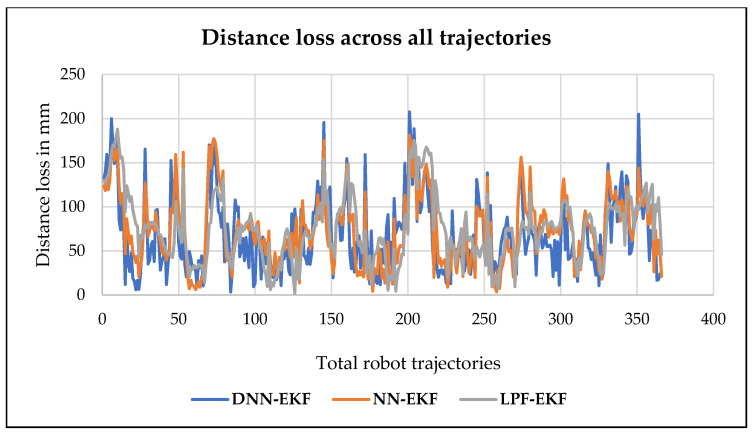
Distance loss among proposed DNN-EKF, NN-EKF, and LPF-EKF.

Figure 9 shows the detailed results of the proposed DNN-EKF approach in comparison with the two other algorithms of NN-EKF and LPF-EKF in terms of distance loss for visualization in a display graph. We are assessing their respective performance in predicting robot trajectories. Distance loss is a critical metric that quantifies the average discrepancy between the predicted trajectories and the actual trajectories of the robot. In simple terms, distance loss measures how far off the robot’s predicted movements are from its actual movements. It calculates the average discrepancy between the positions or trajectories that the robot is predicted to follow and the ones it follows during execution. Predicted trajectories are the robot’s intended or calculated paths based on a model or algorithm, such as neural networks, control systems, or planning software. In this context, the model predicts where the robot is supposed to go. Actual trajectories are the real-world paths that the robot follows, which may deviate from the predictions due to factors like sensor noise, imperfect actuation, or environmental changes. Lower distance loss values indicate higher accuracy and precision in the prediction of robot movement. To display this comparison graphically, we create a graph where the x-coordinate represents the trajectories of algorithms (DNN-EKF, NN-EKF, and LPF-EKF), and the y-coordinate represents the distance loss values in mm. Each algorithm has its respective bar or data point, allowing for a visual comparison of their performance.

In comparing the performance of different localization methods, the average distance loss, which is shown in Table 3, is observed to vary among the proposed models. Specifically, the proposed DNN-EKF approach exhibits the lowest average distance loss at 68.06 mm, outperforming both the NN-EKF method with a loss of 73.35 mm and the LPF-EKF method with a loss of 75.30 mm.

This indicates that the DNN-EKF model achieves superior accuracy in localizing the robot, resulting in a more precise estimation of distance loss compared to the other evaluated approaches. In the context of the comparison, the proposed approach is deemed superior to the method referenced in [31] by a margin of 7.24 mm. Similarly, in comparison to the method referenced in [16], the proposed approach demonstrates a superiority of 5.29 mm. This indicates that the proposed approach outperforms the methods referenced in [16,31] by a higher level of effectiveness or achievement in the evaluated work.

## 5. Conclusions

In conclusion, this paper presents an innovative approach that integrates a deep neural network with an extended Kalman filter (DNN-EKF) to significantly enhance indoor localization accuracy for mobile robots. The results clearly demonstrate that the proposed model outperforms existing models, including NN-EKF, LPF-EKF, and other traditional methods. Specifically, the DNN-EKF method achieved an optimal performance with a learning rate of 0.1, yielding a minimum distance loss of 68.06 mm on average, whereas the NN-EKF model resulted in an average distance loss of 73.35 mm, and LPF-EKF showed an average distance loss of 75.30 mm. These findings underscore the superior effectiveness of the DNN-EKF model in providing precise localization in indoor environments, particularly when using UWB technology. This makes the model highly suitable for real-time applications in robotics, especially in dynamic and noisy settings. Furthermore, the ability of the DNN-EKF to learn complex patterns in sensor data and adapt to varying conditions presents significant advantages over traditional methods. For future work, we will explore the integration of other advanced deep learning models, such as RNNs or LSTM, with EKF to further enhance localization performance. By incorporating these models, we aim to capture more intricate temporal dependencies and improve the robustness of the system. These advancements will allow mobile robots to perform even more effectively in complex, dynamic, real-world environments, paving the way for more accurate, reliable, and adaptive autonomous navigation systems. 

## Figures and Tables

**Table 1:** The values of parameter settings for the proposed model.

Number	Parameter	Value
1	Alpha	0.001
2	Batch size	32
3	Learning rate	$10^{-1},10^{-2},10^{-3}$
4	Number of hidden layers	3
5	Number of neurons per layer	50
6	Episodes	500
7	Activation Function	ReLU
8	Number of iterations	2000

**Table 2:** Mean squared error (MSE) between two sets of values, x- and y-coordinates.

Number	Localization Method	MSE for y-Coordinate	MSE for x-Coordinate
1	DNN-EKF (lr = 0.1)	56.63	56.82
2	DNN-EKF (lr = 0.01)	58.82	56.24
3	DNN-EKF (lr = 0.001)	58.92	56.84
4	LPF-EKF	58.93	59.44
5	NN-EKF	55.16	66.26
6	UWB-based	64.56	60.13

**Table 3:** Average distance loss of the proposed and existing works.

Number	Localization Method	Average Distance Loss
1	DNN-EKF (lr = 0.1)	68.06 mm
2	DNN-EKF (lr = 0.01)	69.02 mm
3	DNN-EKF (lr = 0.001)	69.08 mm
4	LPF-EKF	75.30 mm
5	NN-EKF	73.35 mm
6	UWB-based	76.73 mm

## Data Availability

The raw data supporting the conclusions of this article will be made available by the authors upon request.

## References

[1] Crețu-Sîrcu A.L., Schiøler H., Cederholm J.P., Sîrcu I., Schjørring A., Larrad I.R., Berardinelli G., Madsen O. (2022). Evaluation and Comparison of Ultrasonic and UWB Technology for Indoor Localization in an Industrial Environment. Sensors.

[2] Ye X., Yu Z. Research on UWB positioning method based on deep learning. Proceedings of the 2020 5th International Conference on Mechanical, Control and Computer Engineering (ICMCCE).

[3] Rawat P., Singh K.D., Chaouchi H., Bonnin J.M. (2014). Wireless Sensor Networks: A Survey on Recent Developments and Potential Synergies. J. Supercomput..

[4] Huang B., Zhao J., Liu J. (2019). A Survey of Simultaneous Localization and Mapping with an Envision in 6G Wireless Networks. arXiv.

[5] Liu H., Darabi H., Banerjee P., Liu J. (2007). Survey of Wireless Indoor Positioning Techniques and Systems. IEEE Trans. Syst. Man Cybern. Part C Appl. Rev..

[6] Jan B., Farman H., Javed H., Montrucchio B., Khan M., Ali S. (2017). Energy Efficient Hierarchical Clustering Approaches in Wireless Sensor Networks: A Survey. Wirel. Commun. Mob. Comput..

[7] Horiba M., Okamoto E., Shinohara T., Matsumura K. An Improved NLOS Detection Scheme for Hybrid-TOA/AOA-Based Localization in Indoor Environments. Proceedings of the 2013 IEEE International Conference on Ultra-Wideband (ICUWB).

[8] O’Lone C.E., Dhillon H.S., Buehrer R.M. (2022). Characterizing the First-Arriving Multipath Component in 5G Millimeter Wave Networks: TOA, AOA, and Non-Line-of-Sight Bias. IEEE Trans Wirel. Commun..

[9] Geng M., Wang Y., Tian Y., Huang T. CNUSVM: Hybrid CNN-Uneven SVM Model for Imbalanced Visual Learning. Proceedings of the 2016 IEEE Second International Conference on Multimedia Big Data (BigMM).

[10] Gezici S., Tian Z., Giannakis G.B., Kobayashi H., Molisch A.F., Poor H.V., Sahinoglu Z. (2005). Localization via Ultra-Wideband Radios: A Look at Positioning Aspects for Future Sensor Networks. IEEE Signal Process. Mag..

[11] Oajsalee S., Tantrairatn S., Khaengkarn S. Study of ROS Based Localization and Mapping for Closed Area Survey. Proceedings of the 2019 IEEE 5th International Conference on Mechatronics System and Robots (ICMSR).

[12] Queralta J.P., Martínez Almansa C., Schiano F., Floreano D., Westerlund T. UWB-Based System for UAV Localization in GNSS-Denied Environments: Characterization and Dataset. https://ieeexplore.ieee.org/abstract/document/9341042/.

[13] Sesyuk A., Ioannou S., Raspopoulos M. (2022). A Survey of 3D Indoor Localization Systems and Technologies. Sensors.

[14] Wang Y. (2015). Linear Least Squares Localization in Sensor Networks. EURASIP J. Wirel. Commun. Netw..

[15] Borhan N., Saleh I., Yunus A., Rahiman W., Novaliendry D., Risfendra Reducing UWB Indoor Localization Error Using the Fusion of Kalman Filter with Moving Average Filter. Proceedings of the 2023 IEEE International Conference on Automatic Control and Intelligent Systems (I2CACIS).

[16] Karfakis P.T., Couceiro M.S., Portugal D., Cortesão R. UWB Aided Mobile Robot Localization with Neural Networks and the EKF. Proceedings of the 2022 IEEE International Conference on Systems, Man, and Cybernetics (SMC).

[17] Cano J., Ding Y., Pages G., Chaumette E., Le Ny J. A Robust Kalman Filter Based Approach for Indoor Robot Positionning with Multi-Path Contaminated UWB Data. Proceedings of the ICASSP 2023—2023 IEEE International Conference on Acoustics, Speech and Signal Processing (ICASSP).

[18] Deremetz M., Lenain R., Laneurit J., Debain C., Peynot T. Autonomous Human Tracking using UWB sensors for mobile robots: An Observer-Based approach to follow the human path. Proceedings of the 2020 IEEE Conference on Control Technology and Applications (CCTA).

[19] Nosrati L., Fazel M.S., Ghavami M. (2022). Improving Indoor Localization Using Mobile UWB Sensor and Deep Neural Networks. IEEE Access.

[20] Kulikov R.S., Tsaregorodcev D.V., Petukhov N.I., Chugunov A.A., Mitic A. Algorithm of Related Points Positioning Using Extend Kalman Filter. Proceedings of the 2020 International Youth Conference on Radio Electronics, Electrical and Power Engineering (REEPE).

[21] Zhao Y., Yang Y., Kyas M. Comparing centralized Kalman filter schemes for indoor positioning in wireless sensor network. Proceedings of the 2011 International Conference on Indoor Positioning and Indoor Navigation.

[22] Kolakowski M. Comparison of Extend and Unscented Kalman Filters Performance in a Hybrid BLE-UWB Localization System. Proceedings of the 2020 23rd International Microwave and Radar Conference (MIKON).

[23] Abbas H.A., Boskany N.W., Ghafoor K.Z., Rawat D.B. Wi-Fi Based Accurate Indoor Localization System Using SVM and LSTM Algorithms. Proceedings of the 2021 IEEE 22nd International Conference on Information Reuse and Integration for Data Science (IRI).

[24] Chriki A., Touati H., Snoussi H. SVM-Based Indoor Localization in Wireless Sensor Networks. Proceedings of the 2017 13th International Wireless Communications and Mobile Computing Conference (IWCMC).

[25] Zhou G., Luo J., Xu S., Zhang S., Meng S., Xiang K. (2021). An EKF-Based Multiple Data Fusion for Mobile Robot Indoor Localization. Assem. Autom..

[26] Li J., Gao T., Wang X., Bai D., Guo W. (2022). The IMU/UWB/Odometer Fusion Positioning Algorithm Based on EKF. J. Phys..

[27] Long Z., Xiang Y., Lei X., Li Y., Hu Z., Dai X. (2022). Integrated Indoor Positioning System of Greenhouse Robot Based on UWB/IMU/ODOM/LIDAR. Sensors.

[28] Yi D.-H., Lee T.-J., Cho D.-I. (2018). A New Localization System for Indoor Service Robots in Low Luminance and Slippery Indoor Environment Using A Focal Optical Flow Sensor Based Sensor Fusion. Sensors.

[29] McLoughlin B.J., Pointon H.A.G., McLoughlin J.P., Shaw A., Bezombes F.A. (2018). Uncertainty Characterisation of Mobile Robot Localization Techniques Using Optical Surveying Grade Instruments. Sensors.

[30] Dai Y. (2022). Research on Robot Positioning and Navigation Algorithm Based on SLAM. Wirel. Commun. Mob. Comput..

[31] Ranjan R., Shin D., Jung Y., Kim S., Yun J.-H., Kim C.-H., Lee S., Kye J. (2024). Comparative Analysis of Integrated Filtering Methods Using UWB Localization in Indoor Environment. Sensors.

[32] Krishnan S., Sharma P., Guoping Z., Woon O.H. A UWB based Localization System for Indoor Robot Navigation. Proceedings of the 2007 IEEE International Conference on Ultra-Wideband.

[33] Yang T., Cabani A., Chafouk H. (2021). A Survey of Recent Indoor Localization Scenarios and Methodologies. Sensors.

[34] Wang T., Zhao H., Shen Y. (2020). An Efficient Single-Anchor Localization Method Using Ultra-Wide Bandwidth Systems. Appl. Sci..

[35] Wang Y., Jie H., Cheng L. (2019). A Fusion Localization Method Based on a Robust Extended Kalman Filter and Track-Quality for Wireless Sensor Networks. Sensors.

[36] Mahdi A.A., Chalechale A., Abdelraouf A. (2022). A Hybrid Indoor Positioning Model for Critical Situations Based on Localization Technologies. Mob. Inf. Syst..

[37] Alatise M., Hancke G. (2017). Pose Estimation of a Mobile Robot Based on Fusion of IMU Data and Vision Data Using an Extended Kalman Filter. Sensors.

[38] Xing B., Zhu Q., Pan F., Feng X. (2018). Marker-Based Multi-Sensor Fusion Indoor Localization System for Micro Air Vehicles. Sensors.

[39] Chen L., Hu H., McDonald-Maier K. EKF Based Mobile Robot Localization. Proceedings of the 2012 3rd International Conference on Emerging Security Technologies, EST 2012.

